# Stabilization of phosphofructokinase 1 platelet isoform by AKT promotes tumorigenesis

**DOI:** 10.1038/s41467-017-00906-9

**Published:** 2017-10-16

**Authors:** Jong-Ho Lee, Rui Liu, Jing Li, Chuanbao Zhang, Yugang Wang, Qingsong Cai, Xu Qian, Yan Xia, Yanhua Zheng, Yuji Piao, Qianming Chen, John F. de Groot, Tao Jiang, Zhimin Lu

**Affiliations:** 1 Brain Tumor Center and Department of Neuro-Oncology, The University of Texas MD Anderson Cancer Center, Houston, TX 77030 USA; 2 State Key Laboratory of Oral Diseases, West China Hospital of Stomatology, Sichuan University, Chengdu, Sichuan 610041 China; 3 Beijing Neurosurgical Institute, Capital Medical University, Beijing, 100050 China; 4 Department of Molecular and Cellular Oncology, The University of Texas MD Anderson Cancer Center, Houston, TX 77030 USA; 5 The University of Texas Graduate School of Biomedical Sciences at Houston, Houston, TX 77030 USA

## Abstract

Phosphofructokinase 1 (PFK1) plays a critical role in glycolysis; however, its role and regulation in tumorigenesis are not well understood. Here, we demonstrate that PFK1 platelet isoform (PFKP) is the predominant PFK1 isoform in human glioblastoma cells and its expression correlates with total PFK activity. We show that PFKP is overexpressed in human glioblastoma specimens due to an increased stability, which is induced by AKT activation resulting from phosphatase and tensin homologue (PTEN) loss and EGFR-dependent PI3K activation. AKT binds to and phosphorylates PFKP at S386, and this phosphorylation inhibits the binding of TRIM21 E3 ligase to PFKP and the subsequent TRIM21-mediated polyubiquitylation and degradation of PFKP. PFKP S386 phosphorylation increases PFKP expression and promotes aerobic glycolysis, cell proliferation, and brain tumor growth. In addition, S386 phosphorylation in human glioblastoma specimens positively correlates with PFKP expression, AKT S473 phosphorylation, and poor prognosis. These findings underscore the potential role and regulation of PFKP in human glioblastoma development.

## Abstract (web summary)

Phosphofructokinase 1 (PFK1) plays a critical role in glycolysis. Here the authors show that PFK1 platelet isoform is upregulated in Glioblastoma and is required for tumor growth mechanistically, such upregulation is due to an increased stability induced by AKT activation via phosphorylation on residue S386.

## Introduction

Regardless of extracellular oxygen levels, most cancer cells produce energy predominantly by a high rate of glycolysis, followed by lactic acid fermentation in the cytosol, whereas most normal cells produce energy by a comparatively low rate of glycolysis, followed by oxidation of pyruvate in mitochondria^1^. This metabolic alteration, termed the Warburg effect, provides the high energy and biosynthetic materials required for tumor cell growth^2, 3^.

In the glycolytic pathway, phosphofructokinase 1 (PFK1) catalyzes one of the key regulatory and rate-limiting steps of glycolysis by converting fructose 6-phosphate and ATP to fructose 1,6-bisphosphate and ADP^4^. PFK1 has 3 isoforms: platelet (PFKP), muscle (PFKM), and liver (PFKL)^4, 5^. PFKL is the most abundant in the liver and kidneys, whereas PFKM and PFKP are the only forms present in adult muscles and platelets, respectively. In contrast, all 3 isoforms are present in the brain and other tissues^6–8^. PFK1 is allosterically inhibited by phosphoenolpyruvate, citrate, and ATP and activated by a high concentration of AMP, ADP, and fructose-2,6-bisphosphate (F-2,6-BP)^9^. Of note, PFKP is the prominent PFK1 isoform in breast carcinoma, ascites tumors, and B- and T-cell leukemias, in which total PFK1 expression or activity is upregulated^10–13^. However, the mechanisms underlying the regulation of PFK1 expression in cancer cells still need to be elucidated.

Ubiquitylation and proteasome-dependent degradation are instrumental in the regulation of cell signaling protein expression^14^. Tripartite motif (TRIM)-containing protein 21 (TRIM21), also known as Ro52 or RNF81, is a RING finger domain-containing E3 ligase that belongs to the TRIM superfamily, which has been found to play important roles in innate and acquired immunity^15^. TRIM21 expression, which is significantly increased in the peripheral blood mononuclear cells of patients, is associated with the autoimmune diseases systemic lupus erythematosus and Sjögren’s syndrome and plays a role in the increased apoptosis of circulating leukocytes^16^. TRIM21 is an autoantigen that is recognized by antibodies in the sera of patients with lupus and Sjögren’s syndrome, and anti-TRIM21 antibodies have been used as a diagnostic marker for decades^17^. TRIM21-mediated ubiquitylation and degradation of interferon regulatory transcription factor (IRF)3, IRF5, IRF7, and IRF8 regulate type 1 interferon and cytokine production. TRIM21 is upregulated at the site of autoimmune inflammation and may play an important role in the pathogenesis of autoimmunity^18^. Of note, TRIM21 expression is downregulated in hepatocellular carcinoma cells and is significantly and inversely correlated with patient prognosis, suggesting that TRIM21 acts as a tumor suppressor by inhibiting hepatocellular carcinoma cell proliferation, migration, and invasion^19^. However, the mechanism underlying TRIM21-regulated tumor development is unknown.

In this study, overexpression of PFKP was detected in human glioblastom﻿a (GBM) and resulted from AKT activation that, in turn, was induced by phosphatase and tensin homologue (PTEN) loss and epidermal growth factor receptor (EGFR)-dependent phosphoinositide 3-kinase (PI3K) activation. AKT phosphorylated PFKP at Ser386 and blocked the TRIM21-mediated polyubiquitylation and degradation of PFKP. PFKP S386 phosphorylation promoted glycolysis, cell proliferation, and brain tumor growth.

## Results

### PFKP expression is required for the Warburg effect and brain tumor growth

**Fig. 1:**
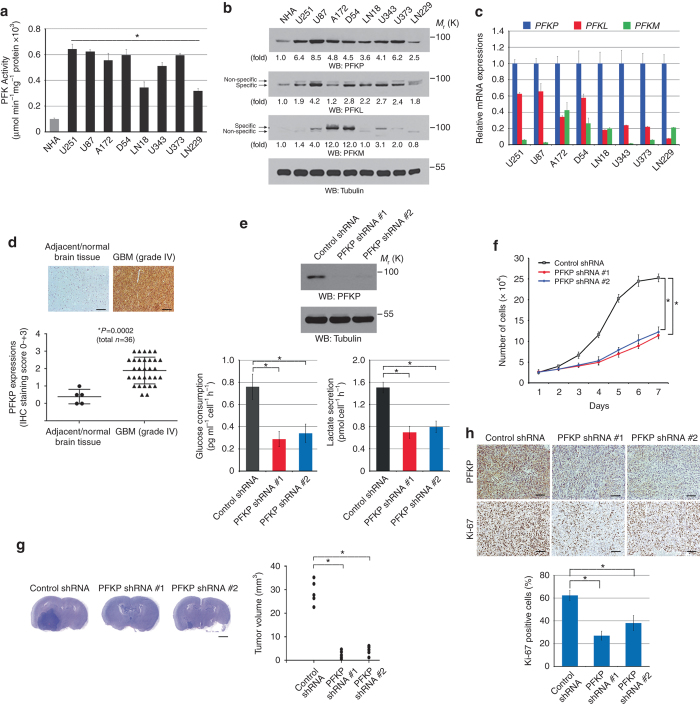
PFKP expression is required for the Warburg effect and brain tumor growth. **a** PFK enzymatic activity was measured in normal human astrocytes (NHA) and the indicated GBM cells. Data represent the means ± s.d. of three independent experiments. *P < 0.001, based on the Student’s *t* test. **b** The protein expression levels of PFK1 isoforms in NHA and the indicated GBM cells were determined by immunoblotting analyses with the indicated antibodies, respectively. **c** Relative mRNA expression levels of PFK1 isoforms were determined. The results were based on those in Supplementary Fig. 1a, c. **d** Microarrays of human GBM and normal brain tissue were immunostained with an anti-PFKP antibody. Representative images are shown (top panel). Data represent the means ± s.d. (bottom panel). *P < 0.001, based on the Student’s *t* test. Scale bar, 100 μm. **e** U87/EGFRvIII cells were transfected with different shRNAs against PFKP. PFKP shRNA#1 was used for the subsequent experiments (top panel). The cells were cultured in no-serum DMEM for 24 h. The media were collected to analyze glucose consumption (bottom left panel) and lactate secretion (bottom right panel). All results were normalized to the final cell number. Data represent the means ± s.d. of three independent experiments. *P < 0.001, based on the Student’s *t* test. **f** Control U87/EGFRvIII cells or PFKP-depleted U87/EGFRvIII cells were cultured in 1% serum medium for the indicated periods of time and harvested for cell counting. Data represent the mean ± s.d. of three independent experiments. *P < 0.001, based on the Student’s *t* test. **g** A total of 5 × 10^5^ control U87/EGFRvIII cells or PFKP-depleted U87/EGFRvIII cells were intracranially injected into athymic nude mice. After 2 weeks, the mice were euthanized and examined for tumor growth. Hematoxylin-and-eosin–stained coronal brain sections show representative tumor xenografts (left panel). Tumor volumes were measured by using length (*a*) and width (*b*) and calculated using the equation *V* = *ab* ^2^/2. Data represent the means ± s.d. of 5 mice (right panel). Note that the scores of some samples overlap. *P < 0.001, based on the Student’s *t*-test. Scale bar, 2 mm. **h** IHC analyses of the tumor tissues were performed with anti-PFKP and anti-Ki-67 antibodies. Representative staining (top panel) and quantification of the staining (bottom panel) are shown. *P < 0.001, based on the Student’s *t*-test. Scale bar, 100 μm

PFK1 catalyzes a rate-limiting step of glycolysis^4^. To determine the role of PFK1 in the Warburg effect, we first examined the total activity of PFK in both normal human astrocytes (NHA) and human glioblastoma (GBM) cell lines. As shown in Fig. 1a, GBM cells exhibited much more PFK activity than did normal astrocytes. Analyses of the isoform expression profile using quantitative real-time PCR and immunoblotting showed that the mRNA levels (Supplementary Fig. 1a) and corresponding protein expression levels (Fig. 1b) of PFK in all examined GBM cell lines were substantially higher than were the levels in NHA, whereas more variable mRNA and protein expression levels of PFKL and PFKM were observed in GBM cell lines. In addition, PFKP levels were elevated in primary GBM cells (Supplementary Fig. 1b). Of note, *PFKP* mRNA expression levels, which were higher than those of *PFKL* and *PFKM* (Fig. 1c, Supplementary Fig. 1c), were the only ones that were correlated with PFK activity (Supplementary Fig. 1d).

In line with these findings, immunohistochemical (IHC) staining of 31 human GBM specimens and 5 normal human brain tissue samples from the same patients or from individuals with no cancer showed that PFKP expression levels in GBM specimens were much higher than those in normal human brain tissue (Fig. 1d). These results strongly suggest that GBM increases PFKP expression and PFK activity. Of importance, depletion of PFKP in U251 (Supplementary Fig. 1e) and U87 human GBM cells that overexpressed constitutively active EGFRvIII mutant (U87/EGFRvIII) (Fig. 1e) revealed that a reduction in PFKP expression impaired glucose uptake, lactate production (Supplementary Fig. 1e and Fig. 1e), and cell proliferation (Supplementary Fig. 1f and Fig. 1f). Consistent with these results, depletion of PFKP inhibited the growth of brain tumors derived from intracranially injected U87/EGFRvIII cells (Fig. 1g) and reduced tumor cell proliferation, as evidenced by the intensity of Ki-67 expression (Fig. 1h). These results indicate that PFKP plays a vital role in the Warburg effect and brain tumor growth.

### AKT activation resulted from PTEN loss and EGFR-dependent PI3K activation induced PFKP upregulation

**Fig. 2:**
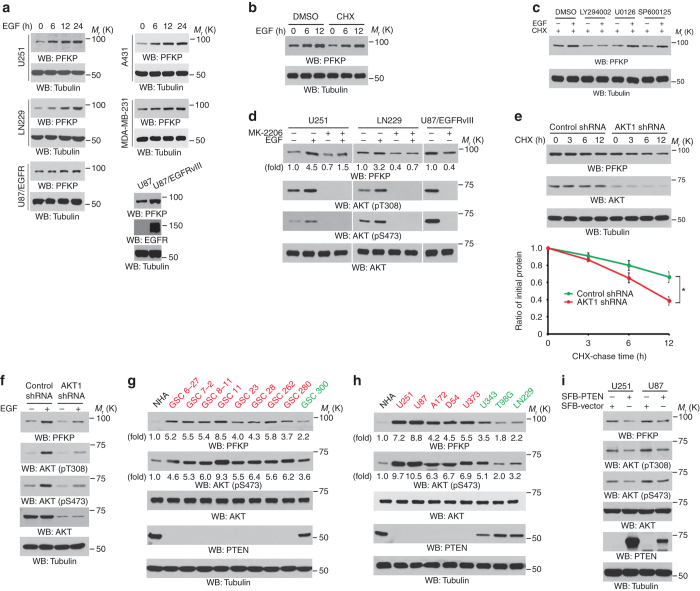
AKT activation resulted from PTEN loss and EGFR-dependent PI3K activation induces PFKP upregulation. Immunoblotting analyses were performed with the indicated antibodies. **a** The indicated tumor cells were serum-starved for 12 h and then stimulated with or without EGF (100 ng ml^−1^) for the indicated periods of time. **b** Serum-starved U251 cells were stimulated with or without EGF (100 ng ml^−1^) for the indicated periods of time in the presence of DMSO or CHX (100 μg ml^−1^). **c** Serum-starved U251 cells were pretreated with CHX (100 μg ml^−1^) for 1 h and then stimulated with EGF (100 ng ml^−1^) for 12 h in the presence or absence of the indicated inhibitors. **d** Serum-starved U251 and LN229 cells were pretreated with DMSO or MK-2206 (5 μM) for 2 h and then stimulated with or without EGF (100 ng ml^−1^) for 24 h. U87/EGFRvIII cells were cultured in non-serum DMEM for 24 h in the presence of DMSO or MK-2206 (5 μM). **e** U87/EGFRvIII cells with stable expression of AKT1 shRNA or a control shRNA were treated with CHX (100 μg ml^−1^) for the indicated periods of time. Quantification of PFKP levels relative to tubulin is shown. Data represent the means ± s.d. of three independent experiments. *P < 0.01, based on the Student’s *t* test. **f** Serum-starved U251 cells with stable expression of AKT1 shRNA or a control shRNA were stimulated with or without EGF (100 ng ml^−1^) for 24 h. **g**, **h** NHA, the indicated primary GBM cells **g**, and established GBM cell lines **h** were subjected to an immunoblotting analysis. The cells with PTEN loss are highlighted in red, and the cells with WT PTEN expression are shown in green. **i** U251 and U87 cells were transfected with SFB-tagged control vector or SFB-PTEN for 48 h

To determine whether the activation of EGFR, which is overexpressed or mutated in many types of cancer^20^, has an effect on PFKP expression, we used EGF to stimulate U251, LN229, and EGFR-overexpressed U87 (U87/EGFR) GBM cells, A431 human epidermoid carcinoma cells, and MDA-MB-231 human breast carcinoma cells. EGF treatment increased the expression of PFKP in a time-dependent manner (Fig. 2a). In addition, expression of EGFRvIII mutant greatly increased PFKP expression in U87 cells (Fig. 2a). To determine whether EGFR activation-enhanced PFKP expression resulted from increased PFKP stability, we pretreated U251 cells with cycloheximide (CHX) to block protein synthesis; this treatment had a limited effect on EGF-induced PFKP expression (Fig. 2b). These results suggest that EGFR activation enhances PFKP expression primarily by enhancing PFKP stability.

To determine how PFKP expression is regulated by EGFR activation, we pretreated U251 cells with the PI3K inhibitor LY294002, MEK inhibitor U0126, and JNK inhibitor SP600125, which successfully blocked EGF-induced AKT, ERK, and c-Jun phosphorylation, respectively (Supplementary Fig. 2a). Inhibition of PI3K/AKT, but not of ERK or JNK, largely abrogated EGF-induced PFKP upregulation in the presence of CHX (Fig. 2c). In line with this result, pretreatment of several types of cancer cells with MK-2206, a selective AKT1/2/3 inhibitor, blocked EGF-stimulated PFKP expression (Fig. 2d and Supplementary Fig. 2b). To test whether other growth factors also regulate PFKP expression, we treated U251 cells with insulin-like growth factor (IGF) and fibroblast growth factor (FGF). As shown in Supplementary Fig. 2c, IGF and FGF treatment activated AKT and increased the expression of PFKP (Supplementary Fig. 2c). In addition, MK-2206 AKT inhibitor treatment greatly blocked the IGF- or FGF-enhanced PFKP expressions. These results suggested that IGF and FGF acting like EGF enhance PFKP expressions.

We next further examined the role of AKT in PFKP expression. A reduction in AKT expression as a result of AKT1 shRNA expression (Fig. 2e) or MK-2206 treatment (Supplementary Fig. 2d) decreased the half-life of endogenous PFKP, while the expression of active Myr-AKT1 prolonged the half-life of PFKP (Supplementary Fig. 2e). Consistent with these results, reduced AKT expression in U251 cells or U87/EGFRvIII cells largely blocked EGF- or EGFRvIII-induced PFKP upregulation (Fig. 2f and Supplementary Fig. 2f). These results indicate that AKT activation is required for EGF-enhanced PFKP stability.

AKT activity is regulated not only by growth factor but also by the PTEN tumor suppressor, which is one of the most frequently altered genes in cancer^21, 22^. PTEN dephosphorylates phosphatidylinositol 3,4,5 trisphosphate (PtdIns(3,4,5) P3), an activator of 3-phosphoinositide-dependent kinase (PDK) and AKT. Loss of PTEN function leads to increased levels of PtdIns(3,4,5)P3 and activation of AKT^23–25^. We found that primary GBM cells (Fig. 2g) or GBM cell lines (Fig. 2h), which lacked PTEN expression resulting from genetic deletions or mutations of PTEN, had higher levels of AKT phosphorylation and PFKP protein expression than did primary GBM cells, several GBM cell lines, or NHA with wild-type (WT) PTEN expression; of note, the levels of AKT phosphorylation were directly correlated with the PFKP protein expression levels. In addition, the levels of WT PTEN expression, with the highest level in NHA, were inversely correlated with the levels of AKT phosphorylation, PFKP expression (Figs. 2g, h), and PFKP half-lives (Supplementary Fig. 2g). Re-expression of WT PTEN in PTEN-deficient U251 and U87 cells inhibited AKT phosphorylation and PFKP expression (Fig. 2i). These results indicate that AKT activation resulted from PTEN loss or EGFR-dependent PI3K activation induces PFKP upregulation.

### AKT binds with and phosphorylates PFKP at Ser386

**Fig. 3:**
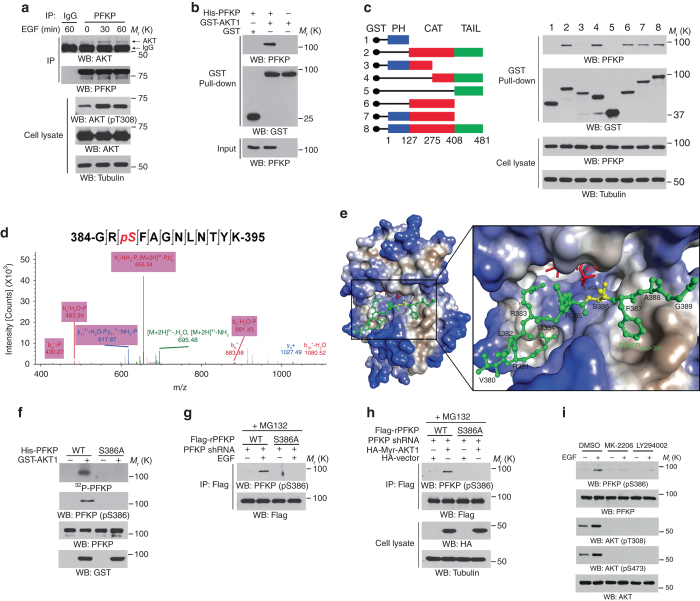
AKT binds with and phosphorylates PFKP at Ser386. Immunoblotting analyses were performed with the indicated antibodies (**a**–**c**, **f**–**i**). MG132 (10 μM) was added to the cells 6 h before harvesting to eliminate the potential effect of proteasomal degradation on PFKP proteins (**g**, **h**). **a** Serum-starved U251 cells were stimulated with or without EGF (100 ng ml^−1^) for the indicated periods of time. Immunoprecipitation analyses were performed with an anti-PFKP antibody. **b** A GST pull-down assay was performed by mixing purified His-PFKP with purified GST or GST-AKT1. **c** A schematic representation of AKT1 full-length and deletion mutants is shown (left). 293 T cells were transfected with the indicated constructs. A GST pull-down assay was performed (right). **d** In vitro kinase assays were performed by mixing purified His-PFKP with or without purified GST-AKT1. A mass spectrometry analysis of a tryptic fragment of PFKP at a mass-to-charge ratio (*m/z*) of 704.32550 (mass error, 1.49 ppm) matched the 2 + charged peptide 384-GRSFAGNLNTYK-395, suggesting that S386 was phosphorylated. The Mascot score was 46, and the expectation value was 0.00058. **e** Electrostatic surface view of AKT1 in complex with unmodified PFKP (380–VRLRGRSFAG-389 aa) peptide. Close-up view shows that PFKP (380–389) peptide fits into the AKT1 catalytic domain. PFKP S386 residue or γ-phosphate of ATP is highlighted in red or yellow, respectively. The structure was obtained from the PDB database: AKT (1O6L). **f** In vitro kinase assays were performed by mixing purified WT His-PFKP or His-PFKP S386A with or without purified GST-AKT1. **g** PFKP-depleted U251 cells were reconstituted with WT Flag-rPFKP or Flag-rPFKP S386A and then stimulated with or without EGF (100 ng ml^−1^) for 60 min. An immunoprecipitation analysis was performed. **h** PFKP-depleted 293 T cells with reconstituted expression of WT Flag-rPFKP or Flag-rPFKP S386A were co-transfected with an HA vector or HA-Myr-AKT1. An immunoprecipitation analysis was performed. **i** Serum-starved U251 cells were pretreated with DMSO, MK-2206 (5 μM), or LY294002 (20 μM) for 2 h and then stimulated with or without EGF (100 ng ml^−1^) for 60 min

To determine the mechanism underlying AKT-regulated PFKP upregulation, we immunoprecipitated PFKP and performed liquid chromatography-tandem mass spectrometry/mass spectrometry (LC-MS/MS) analyses, which revealed that AKT1, AKT2, and AKT3 were PFKP-interacting proteins (Supplementary Fig. 3a). Co-immunoprecipitation assays showed that Flag-PFKP interacted with HA-AKT1/2 in 293T cells (Supplementary Fig. 3b) and that EGF stimulation enhanced the association between endogenous PFKP and endogenous AKT in U251 (Fig. 3a) and U87/EGFR cells (Supplementary Fig. 3c). This finding was further supported by a glutathione *S*-transferase (GST) pull-down assay using purified recombinant GST-AKT1 and His-PFKP proteins, which showed that AKT1 bound to PFKP directly (Fig. 3b). The interacting region of AKT1 was identified by a pulldown of endogenous PFKP by different purified truncation mutants of GST-AKT1: the amino acid 275–408 was involved in the binding of AKT1 to PFKP (Fig. 3c).

To determine whether AKT1 phosphorylates PFKP, we performed an in vitro phosphorylation assay, followed by LC-MS/MS analyses, which showed that purified GST-AKT1 phosphorylated purified His-PFKP at S386 (Fig. 3d). Structural analyses of AKT1 and the PFKP (380–389) peptide containing the AKT1-phosphorylated S386 showed that the PFKP peptide fit well into the AKT1 catalytic domain and that the distance between PFKP S386 residue and the γ-phosphate of ATP1 was approximately 3.45 Å (Fig. 3e). In addition, the S386 is in a consensus AKT phosphorylation motif and is evolutionally conserved in many species (Supplementary Fig. 3d). AKT1-mediated PFKP phosphorylation was further validated by mixing WT PFKP or PFKP S386A mutant with GST-AKT1 in the presence of [γ-^32^P]ATP, revealing that PFKP S386A mutation abolished AKT1-depdendent PFKP phosphorylation and recognition by a specific anti-phospho-PFKP S386 antibody in vitro (Fig. 3f). The specificity of this PFKP pS386 antibody was validated using a phosphorylation-blocking peptide that blocked the detection of PFKP S386 phosphorylation (Supplementary Fig. 3e). In addition, depletion of endogenous PFKP and reconstitution of RNAi-resistant WT Flag-rPFKP or Flag-rPFKP S386A expression in U251 or U87/EGFRvIII cells revealed that the S386A mutation abolished PFKP phosphorylation induced by EGF (Fig. 3g), constitutively active Myr-AKT1 (Fig. 3h), and EGFRvIII (Supplementary Fig. 3f). Furthermore, pretreatment of U251 or U87/EGFRvIII cells with MK-2206 or LY294002 blocked the S386 phosphorylation of PFKP induced by EGF treatment (Fig. 3i) or EGFRvIII expression (Supplementary Fig. 3g). These results indicate that AKT phosphorylates PFKP at S386.

### AKT-dependent PFKP Ser386 phosphorylation blocks the ubiquitylation and degradation of PFKP

**Fig. 4:**
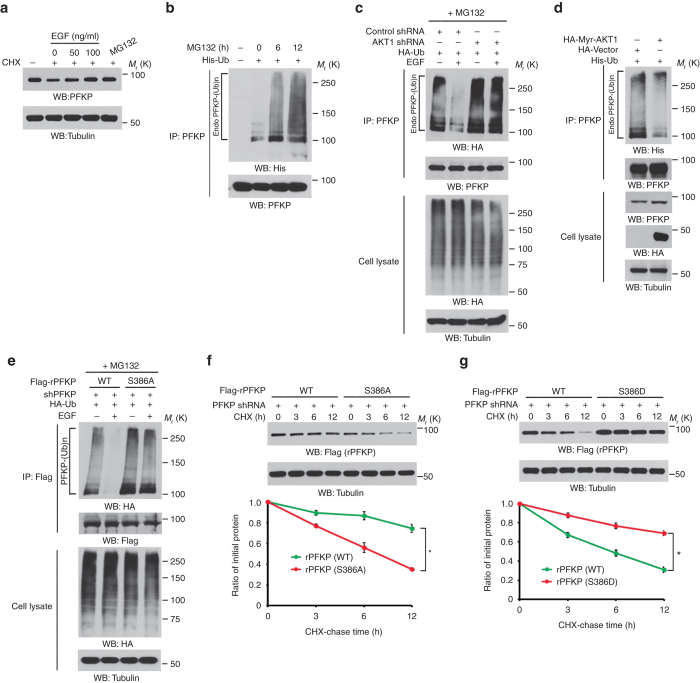
AKT-dependent PFKP Ser386 phosphorylation blocks ubiquitylation and degradation of PFKP. Immunoblotting analyses were performed with the indicated antibodies. **a** Serum-starved U251 cells were stimulated with various dosages of EGF for 12 h in the presence or absence of CHX (100 μg ml^−1^). The proteasome inhibitor MG132 (10 μM) was added to the cells 6 h before cell harvesting. **b** 293T cells were transfected with His-Ub for 24 h and then treated with MG132 (10 μM) for the indicated periods of time. The cells were harvested with a guanidine-HCl-containing buffer. Immunoprecipitation was performed with an anti-PFKP antibody. **c** U251 cells with stable expression of an AKT1 shRNA or a control shRNA were transfected with HA-Ub and then treated with or without EGF (100 ng ml^−1^) for 60 min. MG132 (10 μM) was added to the cells 6 h before they were harvested with a guanidine-HCl-containing buffer. Immunoprecipitation was performed with an anti-PFKP antibody. **d** 293 T cells were co-transfected with HA-vector or HA-Myr-AKT1 and His-Ub. The cells were harvested with a guanidine-HCl-containing buffer. Immunoprecipitation was performed with an anti-PFKP antibody. **e** PFKP-depleted U251 cells with reconstituted expression of WT Flag-rPFKP or Flag-rPFKP S386A mutant were transfected with HA-Ub and then stimulated with or without EGF (100 ng ml^−1^) for 60 min. MG132 (10 μM) was added to the cells 6 h before they were harvested with a guanidine-HCl-containing buffer. Immunoprecipitation of PFKP was performed with an anti-Flag antibody. **f** PFKP-depleted U87/EGFRvIII cells with reconstituted expression of WT Flag-rPFKP or Flag-rPFKP S386A mutant were treated with CHX (100 μg ml^−1^) for the indicated periods of time. The quantification of Flag (rPFKP) levels relative to tubulin levels is shown. Data represent the means ± s.d. of three independent experiments. *P < 0.01, based on the Student’s *t* test. **g** PFKP-depleted 293 T cells with reconstituted expression of WT Flag-rPFKP or Flag-rPFKP S386D mutant were treated with CHX (100 μg ml^−1^) for the indicated periods of time. The quantification of Flag (rPFKP) levels relative to tubulin levels is shown. Data represent the means ± s.d. of three independent experiments. *P < 0.01, based on the Student’s *t* test

To determine the mechanism underlying the activation of EGFR and AKT-induced PFKP upregulation, we treated U251 cells with the proteasome inhibitor MG132 or EGF, which enhanced PFKP expression (Fig. 4a) and PFKP ubiquitylation (Fig. 4b). EGF treatment of U251 cells inhibited PFKP ubiquitylation, and this effect was reversed by AKT depletion (Fig. 4c), which by itself sufficiently induced PFKP ubiquitylation in U87/EGFRvIII cells (Supplementary Fig. 4a). Similar to EGF treatment, overexpression of constitutively active Myr-AKT1 inhibited PFKP ubiquitylation (Fig. 4d). Of note, PFKP S386A mutant had a higher ubiquitylation level than did its WT counterpart in U251 cells and was resistant to EGF-induced inhibition of PFKP ubiquitylation (Fig. 4e). In line with this finding, the half-life of PFKP S386A mutant was much shorter than that of its WT counterpart (Fig. 4f), whereas PFKP S386D phosphorylation-mimic mutant had a much lower turnover rate than did its WT counterpart (Fig. 4g). We next depleted endogenous PFKP in U251 cells and reconstituted the expression of WT Flag-rPFKP, Flag-rPFKP S386A, or Flag-rPFKP S386D in these cells. As expected, EGF treatment enhanced the expression of WT Flag-rPFKP, but not Flag-rPFKP S386A or Flag-rPFKP S386D. In addition, AKT inhibition blocked EGF-enhanced expression of WT Flag-rPFKP without altering expression of Flag-rPFKP S386A or Flag-rPFKP S386D (Supplementary Fig. 4b). These results indicate that EGFR activation-induced and AKT-dependent PFKP Ser386 phosphorylation blocks the ubiquitylation and degradation of PFKP.

### TRIM21 mediates the polyubiquitylation and degradation of PFKP

**Fig. 5:**
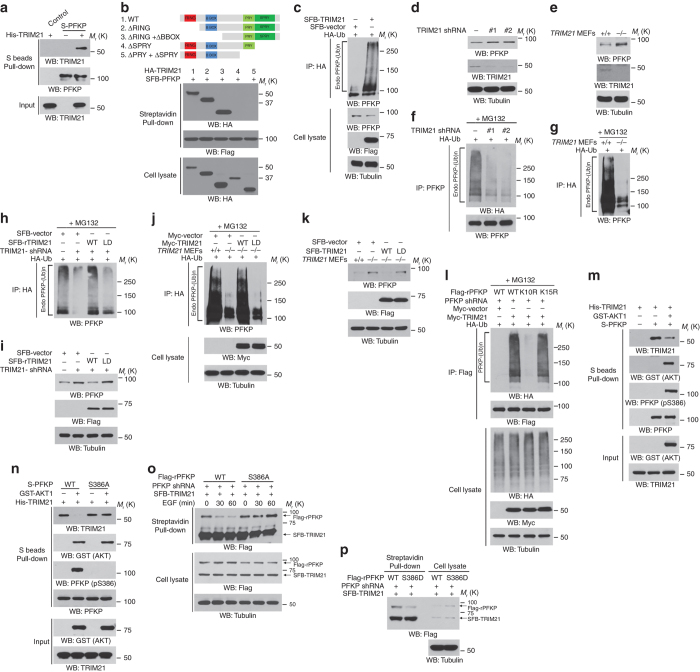
TRIM21 mediates the polyubiquitylation and degradation of PFKP. Immunoblotting analyses were performed with the indicated antibodies. **a** Purified bacterially expressed S-tagged PFKP protein was incubated with purified His-TRIM21. A pull-down assay was performed. **b** Full-length TRIM21 and a series of TRIM21 mutants with deletion (Δ) of various domains (top panel). 293 T cells were co-transfected with SFB-PFKP and WT HA-TRIM21 or their truncation mutants for 24 h. A pull-down assay was performed. **c** 293T cells were co-transfected with SFB (S protein, FLAG, and streptavidin- binding peptide)-tagged TRIM21 and HA-Ub. The cells were harvested with a guanidine-HCl-containing buffer. Immunoprecipitation with an anti-HA antibody was performed. **d** 293 T cells were stably expressed with the TRIM21 shRNAs or a control shRNA. TRIM21 shRNA#1 was used for the subsequent experiments. **e** An analysis of PFKP protein levels in *TRIM21* ^+/+^ and *TRIM21* ^−/−^ MEF cells was performed using immunoblotting analyses. **f** 293T cells with stable expression of the indicated TRIM21 shRNAs or a control shRNA were transfected with HA-Ub. MG132 (10 μM) was added to the cells 6 h before they were harvested with a guanidine-HCl-containing buffer. Immunoprecipitation with an anti-PFKP antibody was performed. **g** *TRIM21* ^+/+^ and *TRIM21* ^−/−^ MEF cells were transfected with HA-Ub. MG132 (10 μM) was added to the cells 6 h before they were harvested with a guanidine-HCl-containing buffer. Immunoprecipitation was performed with an anti-HA antibody. **h** 293T cells with stable expression of the TRIM21 shRNA or a control shRNA, with or without reconstituted expression of WT SFB-rTRIM21 or SFB-rTRIM21 ligase-dead (LD) mutant was transfected with HA-Ub. MG132 (10 μM) was added to the cells 6 h before they were harvested with a guanidine-HCl-containing buffer. Immunoprecipitation was performed with an anti-HA antibody. **i** 293 T cells with stable expression of the TRIM21 shRNA or a control shRNA were reconstituted with WT SFB-rTRIM21 or SFB-rTRIM21 LD mutant. **j** *TRIM21* ^+/+^ and *TRIM21* ^−/−^ MEF cells, with or without reconstituted expression of WT Myc-TRIM21 or Myc-TRIM21 LD mutant, were transfected with HA-Ub. MG132 (10 μM) was added to the cells 6 h before they were harvested with a guanidine-HCl-containing buffer. Immunoprecipitation was performed with an anti-HA antibody. **k** *TRIM21* ^+/+^ and *TRIM21* ^−/−^ MEF cells were transfected with or without WT SFB-TRIM21 or SFB-TRIM21 LD mutant for 48 h. **l** PFKP-depleted 293T cells with reconstituted expression of WT Flag-rPFKP, Flag-rPFKP K10R mutant, or Flag-rPFKP K15R mutant were co-transfected with Myc-tagged TRIM21 and HA-Ub. MG132 (10 μM) was added to the cells 6 h before they were harvested with a guanidine-HCl-containing buffer. Immunoprecipitation was performed with an anti-Flag antibody. **m** An in vitro kinase assay was performed by mixing purified bacterially expressed S-tagged PFKP with or without active GST-AKT1, followed by incubation with purified His-TRIM21 for a pull-down assay. **n** In vitro kinase assays were performed by mixing purified bacterially expressed S-tagged WT PFKP or PFKP S386A mutant with or without purified active GST-AKT1, followed by incubation with purified His-TRIM21 for a pull-down assay. **o** PFKP-depleted U251 cells with reconstituted expression of WT Flag-rPFKP or Flag-rPFKP S386A mutant were transfected with SFB-TRIM21 and then stimulated with or without EGF (100 ng ml^−1^) for the indicated periods of time. A pull-down assay was performed. **p** PFKP-depleted 293 T cells with reconstituted expression of WT Flag-rPFKP or Flag-rPFKP S386D mutant were transfected with SFB-TRIM21. A pull-down assay was performed

We next determined the E3 ligase that was responsible for PFKP degradation. A mass spectrometry analysis of PFKP immunoprecipitates from U251 cells revealed that TRIM21 is a PFKP-associated protein (Supplementary Fig. 3a). A pulldown assay was performed by mixing purified bacterially expressed S-tagged PFKP protein and purified His-TRIM21 and revealed that these 2 proteins directly interacted with each other (Fig. 5a). The expression of a series of TRIM21 truncation mutants with deletion (Δ) of various domains in 293T cells revealed that the deletion of the C-terminal SPRY domain abolished the binding of TRIM21 to PFKP (Fig. 5b), indicating that the TRIM21 SPRY domain plays an essential role in this protein-protein interaction.

To determine whether TRIM21 directly ubiquitylated PFKP, we performed an in vitro ubiquitylation assay and showed that PFKP is ubiquitylated by WT TRIM21, but not a truncated TRIM21 mutant that lacked the RING domain (ΔRING; Supplementary Fig. 5a). In addition, overexpression of TRIM21 resulted in increased ubiquitylation (Fig. 5c), dosage-dependent degradation (Supplementary Fig. 5b), and PFKP turnover rates (Supplementary Fig. 5c) in 293T cells. Of note, TRIM21 overexpression-induced PFKP ubiquitylation was inhibited by the expression of HA-ubiquitin (Ub) K48R but not K63R (Supplementary Fig. 5d), which renders ubiquitin unable to form poly-ubiquitin chains via lysine 48 or 63 linkages with other ubiquitin molecules, respectively. These results show that TRIM21 regulates PFKP degradation through K48-dependent ubiquitylation. In contrast to the effects induced by TRIM21 overexpression, depletion (Fig. 5d) or deficiency of TRIM21 (Fig. 5e), which did not affect PFKP mRNA expression (Supplementary Fig. 5e, f), resulted in upregulation of PFKP (Fig. 5d, e), with a corresponding decrease in ubiquitylation (Fig. 5f, g) and turnover rate (Supplementary Fig. 5g, h) of PFKP in 293T cells and *TRIM21* ^−/−^ mouse embryonic fibroblasts (MEFs). In addition, the effect of TRIM21 depletion (Fig. 5h, i) or deficiency (Fig. 5j, k) on the ubiquitylation and degradation of PFKP was abrogated by the reconstituted expression of WT TRIM21 but not by its ligase-dead mutant (C16A, C31A, and H33W)^15^, indicating that E3 ligase activity of TRIM21 is required for PFKP ubiquitylation and degradation.

To identify the ubiquitylation residue of PFKP, we analyzed the PFKP sequence using the webtool UbPred: predictor of protein ubiquitylation sites (http://www.ubpred.org/) and found that the K10 may have been ubiquitylated (Supplementary Table 1). The PFKP K10R mutant showed complete resistance to TRIM21-mediated ubiquitylation (Fig. 5l), with a much longer half-life than that of its WT counterpart (Supplementary Fig. 5i). These results strongly suggest that TRIM21 polyubiquitylates PFKP at K10 for PFKP degradation.

To determine the role of AKT activation in the regulation of TRIM21-mediated PFKP degradation, we performed an in vitro binding assay in the presence or absence of AKT1 and revealed that AKT1-mediated PFKP phosphorylation greatly reduced the binding of purified TRIM21 to purified WT PFKP (Fig. 5m) but not to purified PFKP S386A (Fig. 5n). Similarly, EGF stimulation disrupted the association of TRIM21 with WT PFKP but not with PFKP S386A (Fig. 5o). MK-2206 treatment inhibited the EGF-reduced interaction between TRIM21 and endogenous PFKP in U251 cells and increased this association in U87/EGFRvIII cells (Supplementary Fig. 5j, k). In line with the results of decreased turnover of PFKP S386D (Fig. 4g), reconstituted expression of PFKP S386D in endogenous PFKP-depleted U251 cells exhibited reduced binding to TRIM21 in contrast to its WT counterpart (Fig. 5p). These results indicate that EGFR activation-induced and AKT-dependent PFKP S386 phosphorylation disassociates TRIM21 from its binding to PFKP.

### PFKP S386 phosphorylation promotes glycolysis, cell proliferation, and tumor growth

**Fig. 6:**
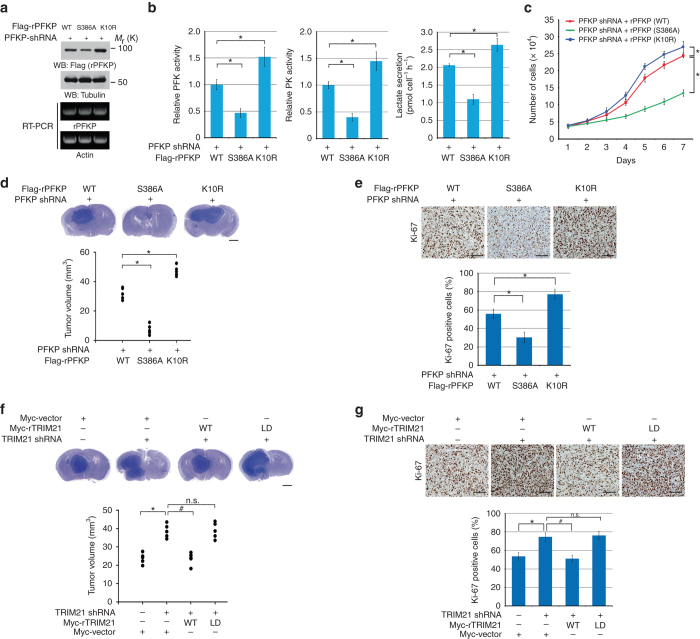
PFKP S386 phosphorylation promotes glycolysis and tumor growth. **a** PFKP-depleted U87/EGFRvIII cells were reconstituted with the indicated protein expression. Immunoblotting analyses were performed with the indicated antibodies (top panel). RT-PCR was performed with the indicated primers to show relatively equal expression of the indicated mRNAs (bottom panel). **b**, **c** PFKP-depleted U87/EGFRvIII cells with reconstituted expression of the indicated proteins were cultured in non-serum DMEM for 24 h (**b**) or in 1% serum medium for the indicated periods of time and harvested for cell counting (**c**). The cells and the media were collected to analyze glucose consumption, PFK activity, PK activity, or lactate secretion. All results were normalized to the final cell number (**b**). Data represent the means ± s.d. of three independent experiments. *P < 0.001, based on the Student’s *t* test. **d** A total of 5 × 10^5^ PFKP-depleted U87/EGFRvIII cells with reconstituted expression of the indicated proteins (**d**) or U87/EGFRvIII cells with or without TRIM21 depletion and reconstituted expression of WT Myc-rTRIM21 or Myc-rTRIM21 LD (**f**) was intracranially injected into athymic nude mice. After 2 weeks, the mice were euthanized and examined for tumor growth. Hematoxylin-and-eosin-stained coronal brain sections show representative tumor xenografts (top panel). Tumor volumes were measured by using length (*a*) and width (*b*) and calculated using the equation *V* = *ab* ^2^/2. Data represent the means ± s.d. of 5 mice (bottom panel). *P < 0.001, based on the Student’s *t*-test **d**. *P < 0.001, ^#^ *P* < 0.001, based on the one-way ANOVA; n.s., not significant **f**. Note that the scores of some samples overlap. Scale bar, 2 mm. **e** IHC analyses of the tumor tissues were performed with an anti-Ki-67 antibody. Representative staining (top panel) and quantification of the staining (bottom panel) are shown. *P < 0.001, based on the Student’s *t* test. Scale bar, 100 μm. **g** IHC analyses of the tumor tissues were performed with an anti-Ki-67 antibody. Representative staining (top panel) and quantification of the staining (bottom panel) are shown. *P < 0.001, ^#^ *P* < 0.001, based on the one-way ANOVA. Scale bar, 100 μm

PFK1 is critical for glycolysis, and fructose 1,6-bisphosphate is an activator of pyruvate kinase M2 (PKM2)^4^. Depletion of endogenous PFKP and reconstitution of WT rPFKP, rPFKP S386A, or rPFKP K10R expression in U87/EGFRvIII revealed a reduction in rPFKP S386A expression and enhanced rPFKP K10R expression compared to the expression of their WT counterpart, although the mRNA expression levels were comparable (Fig. 6a). Similar to the pattern of rPFKP protein expression levels, PFK activity, pyruvate kinase activity, lactate production (Fig. 6b), cell proliferation (Fig. 6c), brain tumor growth in mice (Fig. 6d), and Ki-67 expression levels in brain tumor tissues (Fig. 6e) were reduced by rPFKP S386A but enhanced by rPFKP K10R expression in U87/EGFRvIII cells. An in vitro PFKP activity assay showed that WT rPFKP, rPFKP S386A, and rPFKP K10R had comparable activity (Supplementary Fig. 6a), suggesting that PFKP S386 phosphorylation and PFKP K10 ubiquitylation, which do not change the enzymatic activity of PFKP, regulate PFKP protein expression and thus glycolysis, cell proliferation, and brain tumor development.

To determine the cellular functions of TRIM21, we depleted it in U87/EGFRvIII (Supplementary Fig. 6b) cells. TRIM21 depletion enhanced PFK activity, pyruvate kinase activity, lactate production (Supplementary Fig. 6c), cell proliferation (Supplementary Fig. 6d), and brain tumor growth in mice (Fig. 6f) and Ki-67 expression levels in human brain tumor tissues (Fig. 6g), and these effects were abrogated by reconstituted expression of WT rTRIM21 but not by that of its ligase-dead mutant. These results strongly suggest that TRIM21 negatively regulates glycolysis and tumor cell proliferation and brain tumor formation by regulating PFKP stability.

### PFKP S386 phosphorylation was correlated with PFKP expression and poor GBM patient prognosis

**Fig. 7:**
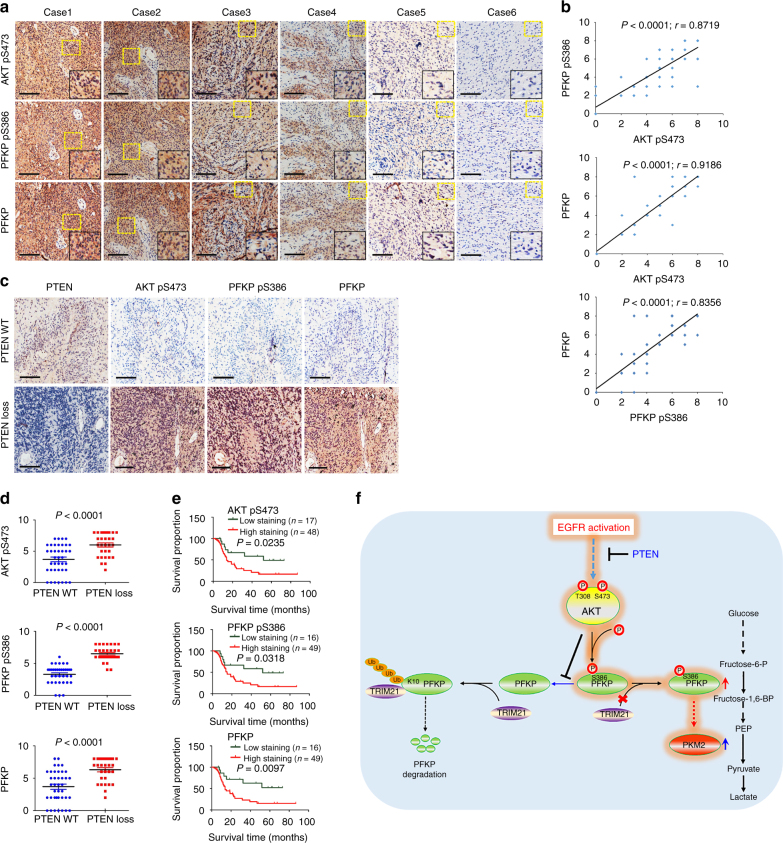
PFKP S386 phosphorylation correlates with PFKP expression and AKT S473 phosphorylation in GBM specimens and with poor prognosis. **a** IHC staining of 65 human GBM specimens was performed with the indicated antibodies. Representative images from the staining of six different specimens are shown. High-magnification images correspond to the areas marked by yellow dotted lines. Scale bar, 100 μm. **b** The IHC stains were scored, and the correlation analyses were performed. Pearson correlation test was used. Note that the scores of some samples overlap. **c**, **d** The inverse correlation between PTEN and AKT pS473, PFKP pS386, or PFKP expression in human GBM specimens was analyzed. The 65 human GBM specimens were classified into 2 groups on the basis of PTEN levels (PTEN WT, *n* = 35; PTEN loss, *n* = 30). PTEN information was obtained by Sanger sequencing covering exon regions of PTEN gene or IHC staining. In IHC staining, tumors with PTEN expression less than 10% of the rate found in WT PTEN tumors were classified as having PTEN loss. Representative images (**c**) and whisker plots (**d**, Student’s *t* test) are shown. Scale bar, 100 μm. **e** Kaplan–Meier plots of the overall survival rates in human GBM specimens (*n* = 65) in the groups with high (staining score, 4–8) and low (staining score, 0–3) expression of AKT pS473, PFKP pS386, and PFKP. The *P* values were calculated using the log-rank test. **f** A schematic of AKT-regulated PFKP phosphorylation and glycolysis

To determine the clinical significance of AKT-mediated PFKP phosphorylation and stability, we analyzed 65 human primary GBM specimens with a specificity-validated antibody (Supplementary Fig. 7). AKT phosphorylation levels were positively correlated with the S386 phosphorylation and expression levels of PFKP (Fig. 7a). Quantification of staining showed that these correlations were significant (Fig. 7b). In addition, AKT phosphorylation and PFKP S386 phosphorylation and PFKP expression levels were inversely correlated with PTEN expression levels (Fig. 7c, d), suggesting that PTEN inhibits PFKP S386 phosphorylation and reduces PFKP stability through inhibition of AKT.

We next compared the survival durations of the 65 patients, all of who had undergone standard adjuvant radiotherapy after surgical resection of GBM, followed by treatment with an alkylating agent (temozolomide in most cases). The median survival durations were 124.1, 167.2, and 131.4 weeks for patients whose tumors had low AKT phosphorylation levels and low PFKP S386 phosphorylation and expression levels, respectively, and 47.4, 49.3 and 49.3 weeks for those whose tumors had high AKT phosphorylation levels and high PFKP S386 phosphorylation and expression levels, respectively (Fig. 7e). These results indicate that AKT-phosphorylated PFKP plays a role in the clinical behavior of human GBM and reveal a correlation among AKT phosphorylation levels, PFKP S386 phosphorylation and expression levels, and the clinical aggressiveness of GBM.

## Discussion

The Warburg effect is critical for tumor development^1, 26^. PFK1 is a rate-limiting enzyme in glycolysis. We demonstrated that PFKP was the primarily expressed PFK1 isoform compared to PFKL and PFKM and was correlated with total PFK activity in human GBM cells. In contrast to normal human astrocytes and normal human brain tissue, PFKP was overexpressed in human GBM cell lines, primary GBM cells, and GBM specimens. In addition, AKT activation, which was induced by PTEN loss and EGFR-dependent PI3K activation, increased PFKP expression by inhibiting proteasomal degradation of PFKP. AKT, which directly bound to PFKP in vitro and formed a complex with PFKP in GBM cells, phosphorylated PFKP at S386. AKT-mediated PFKP S386 phosphorylation inhibited the binding of TRIM21 E3 ligase to PFKP and the subsequent TRIM21-mediated polyubiquitylation and degradation of PFKP. PFKP S386 phosphorylation enhanced PFKP expression, pyruvate kinase activity, lactate production, cell proliferation, and brain tumor growth (Fig. 7f). The clinical significance of AKT-mediated PFKP S386 phosphorylation and the increased stability were evidenced by the positive correlation between AKT S473 phosphorylation and S386 phosphorylation and PFKP expression in human GBM specimens and the positive correlation between these molecular markers and poor survival in GBM patients.

PKM2 is overexpressed in many types of cancer, including GBM, and promotes the Warburg effect^1, 26–28^. In response to EGFR activation, a portion of PKM2 translocates into the nucleus and increases β-catenin-dependent c-Myc expression, which in turn enhances expression of Glut1, lactate dehydrogenase A (LDHA) and PKM2 itself. The highly expressed these glycolytic genes enhance glucose uptake and lactate production^20, 26, 29^. In addition, EGFR activation promotes the translocation of phosphoglycerate kinase 1 (PGK1) into mitochondria, where PGK1 phosphorylates and activates pyruvate dehydrogenase kinase (PDHK), leading to inhibited pyruvate dehydrogenase-dependent mitochondrial pyruvate consumption and enhanced lactate production^30, 31^. PFK1 catalyzes fructose 6-phosphate and ATP into fructose 1,6-bisphosphate and ADP^4^. Fructose 1,6-bisphosphate is an allosteric activator of PKM2^1, 9^. We revealed that AKT-mediated PFKP S386 phosphorylation increased PFKP stability and pyruvate kinase activity. The reconstituted expression of PFKP S386A mutant reduced pyruvate kinase activity, suggesting that PFKP phosphorylation regulates not only its own protein expression but also the activity of PKM2 by regulating the production of its upstream substrate phosphoenolpyruvate, as well as that of its allosteric activator fructose 1,6-bisphosphate.

AKT is activated by EGFR activation and loss of PTEN function^21, 22^. EGFR overexpression and mutation and loss of PTEN function are frequently observed in human cancers. PTEN expression levels were inversely correlated with AKT S473 phosphorylation and PFKP S386 phosphorylation and expression levels, highlighting the importance of the effect of loss of PTEN function on PFKP expression and aerobic glycolysis. These findings underscore the potential of PFKP as a molecular target for the treatment of human cancer.

## Methods

### Materials

Rabbit polyclonal antibody that recognizes PFKP (pS386) was customized from Signalway Biotechnology (Pearland, TX). A peptide containing PFKP pS386 was injected into rabbits. The rabbit serum was collected and purified using an affinity column conjugated with non-phosphorylated PFKP S386 peptide to exclude the antibodies recognizing non-phosphorylated PFKP, followed by an affinity column conjugated with phosphorylated PFKP pS386 peptide to bind to and purify the PFKP pS386 antibody. The PFKP pS386 antibody was then eluted and concentrated. A working concentration of 1 and 5 μg ml^−1^ was used for immunoblotting and immunohistochemical staining, respectively.

Normal rabbit immunoglobulin (sc-2027), polyclonal antibody for mouse TRIM21(sc-21367, clone M-20, 1:1000 for immunoblotting), and monoclonal antibodies for PFKM (sc-67028, 1:1000 for immunoblotting), c-Jun (pS63, sc-822, clone KM-1, 1:1000 for immunoblotting), c-Jun (sc-1694, clone H-79, 1:1000 for immunoblotting), GST (sc-138, clone B-14, 1:1000 for immunoblotting), and Myc (sc-40, clone 9E10, 1:1000 for immunoblotting) were purchased from Santa Cruz Biotechnology (Santa Cruz, CA). Rabbit polyclonal antibodies that recognize human PFKP (12746, 1:1000 for immunoblotting, 1:500 for immunoprecipitation), PFKL(8175, 1:1000 for immunoblotting), AKT (pT308, 4056, 1:1000 for immunoblotting), AKT (pS473, 4060, 1:1000 for immunoblotting), AKT (9272, 1:1000 for immunoblotting), PTEN (9559, 1:1000 for immunoblotting), EKR1/2 (pT202/pY204, 9101, 1:1000 for immunoblotting), and EKR1/2 (9102, 1:1000 for immunoblotting) were purchased from Cell Signaling Technology (Danvers, MA). Mouse monoclonal antibodies for FLAG (F3165, clone M2, 1:5000 for immunoblotting, 1:1000 for immunoprecipitation), His (H1029, clone HIS-1, 1:5000 for immunoblotting), HA (H6908, 1:5000 for immunoblotting, 1:1000 for immunoprecipitation) and tubulin (T6074, clone B-5-1-2, 1:5000 for immunoblotting) were purchased from Sigma (St. Louis, MO). Monoclonal antibody for mouse PFKP (ab137636, 1:1,000 for immunoprecipitation) and polyclonal antibody for human TRIM21 (ab91423, 1:1,000 for immunoprecipitation) were obtained from Abcam (Cambridge, MA). Human recombinant EGF (01-407), IGF (GF306), and FGF (GF003) and an anti-Ki67 (AB9260, 1:300 for immunohistochemistry) antibody were obtained from EMD Millipore (Billerica, MA). Hygromycin (400053), puromycin (540222), and G418 (345810) were purchased from EMD Biosciences (San Diego, CA). Calf intestinal alkaline phosphatase (M0290) was obtained from New England Biolabs (Ipswich, MA). Active GST-AKT1 (A16-10G) was obtained from Signalchem (Richmond, BC, Canada). Recombinant human TRIM21 (pro-328) was obtained from BIOTREND Chemicals (Destin, FL). HyFect transfection reagents (E2650) were obtained from Denville Scientific (Metuchen, NJ). MG132 (1211877-36-9) and CHX (66-81-9) were purchased from Sigma (St. Louis, MO). LY294002 (L-7988) and SP600125 (S-7979) were purchased from LC Laboratories (Woburn, MA). MK-2206 (S1078) was purchased from Selleck Chemicals (Houston, TX).

### Cell culture and transfection

A431, MDA-MB-231, NHA, and GBM cells including U251, U87, A172, D54, LN229, U343, U373, and T98G were obtained from ATCC and are routinely tested for mycoplasma. U87 and U251 cell lines in the experiments were authenticated using short tandem repeat profiling in The University of Texas MD Anderson Cancer Center. Tumor cells including EGFRvIII-overexpressing U87 (U87/EGFRvIII) and *TRIM21* ^−/−^ and *TRIM21* ^+/+^ MEFs were maintained in Dulbecco’s modified Eagle’s medium (DMEM) supplemented with 10% bovine calf serum (HyClone, Logan, UT). Human primary GBM cells were maintained in DMEM/F-12 50/50 supplemented with B27, EGF (10 ng ml^−1^), and basic fibroblast growth factor (10 ng ml^−1^). Cells were plated at a density of 4 × 10^5^ per 60-mm dish or 1 × 10^5^ per well of a six-well plate 18 h before transfection. The transfection procedure was performed as previously described^32^.

### DNA constructs and mutagenesis

PCR-amplified human PFKP, PTEN, and TRIM21 were cloned into pcDNA3.1/hygro(+)-Flag or Myc, pCDH-CMV-MCS-EF1-Puro-SFB, or pET32a vector. pECE-Myr-HA-AKT1(delta4-129) was purchased from Addgene (Cambridge, MA). pcDNA3.1/hygro(+)-Flag PFKP S386A, PFKP S386D, PFKP K10R, PFKP K15R, and pCDH-CMV-MCS-EF1-Puro-SFB TRIM21 LD (C16A, C31A, and H33W) were created using the QuikChange site-directed mutagenesis kit (Stratagene, La Jolla, CA). shRNA-resistant (r) PFKP contained a448c, g450c, c453t, and c456g mutations. shRNA-resistant (r) TRIM21 contained c888a, t891c, and g894a mutations.

The following pGIPZ shRNAs were used: control shRNA oligonucleotide, 52-GCTTCTAACACCGGAGGTCTT-32; PFKP shRNA oligonucleotide, 5′-AGGAACGGCCAGATCGATA-32; AKT1 shRNA oligonucleotide, 5′-TTCTTGAGGAGGAAGTAGC-3′; TRIM21 shRNA#1 oligonucleotide, 5′-AGTATCAGCCACGGATTGG-3′; and TRIM21 shRNA#2 oligonucleotide, 5′-TCCAGAGTGAAAGTGCTGG-3′.

### Reverse transcription and PCR analysis

Total RNA isolation, reverse transcription (RT), and real-time PCR were conducted as described previously^29^. The following primer pairs were used for quantitative real-time PCR: human PFKP, 5′-CGGAAGTTCCTGGAGCACCTCTC-3′ (forward) and 5′-AAGTACACCTTGGCCCCCACGTA-3′ (reverse); human PFKL, 5′-GGCATTTATGTGGGTGCCAAAGTC-3′ (forward) and 5′-CAGTTGGCCTGCTTGATGTTCTCA-3′ (reverse); human PFKM, 5′-GAGTGACTTGTTGAGTGACCTCCAGAAA-3′ (forward) and 5′-CACAATGTTCAGGTAGCTGGACTTCG-3′ (reverse); and β-actin, 5′-ATGGATGACGATATCGCTGCGC-3′ (forward) and 5′-GCAGCACAGGGTGCTCCTCA-3′ (reverse). The following primer pairs were used for RT-PCR: Flag-tagged PFKP, 5′-ATGGACTACAAGGACGACGATGAC-3′ (forward) and 5′- TGGTCATGTCGGTGCCGCAGAA-3′ (reverse).

### Purification of recombinant proteins

His-PFKP WT and His-PFKP S386A were expressed in bacteria and purified^33^. Briefly, the pCold His-PFKP WT and pCold His-PFKP S386A were transformed into BL21/DE3 bacteria. Transformants were used to inoculate 50 ml cultures of LB/ampicillin, which were grown overnight at 37°C to stationary phase. A measure of 5 ml preculture was then used to inoculate 200 ml LB/ampicillin. The cultures were grown at 37°C to an attenuance of approximately 0.4–0.6 at 600 nm before inducing with 0.5 mM IPTG at 16°C for 24 h. Cell pellets were collected, resuspended in 10 ml Bugbuster protein extraction reagent (EMD) with the addition of 20 μl protease cocktail inhibitor (EMD), and incubated at room temperature for 20 min, before centrifuging at 10 000 r.p.m. for 10 min at 4°C. Cleared lysates were then bound to Ni-NTA His bind resin (EMD) for 3 h, with rolling at 4°C. Beads were washed extensively with the extraction buffer before eluting for 1 h in extraction buffer (pH 7.5) plus 500 mM imidazole. Eluted proteins were then dialyzed extensively against 20 mM Tris-HCl pH 8.0, 50 mM NaCl, 10% glycerol and 1 mM dithiothreitol.

### In vitro kinase assay

The GST-AKT1 (500 ng) was incubated with His-PFKP (200 ng) in 25 μl of kinase buffer (50 mM Tris-HCl [pH7.5], 100 mM KCl, 50 mM MgCl_2_, 1 mM Na_3_VO_4_, 1 mM DTT, 5% glycerol, 0.5 mM ATP, and 10 mCi [γ-^32^P]ATP) at 25°C for 1 h. The reaction was terminated by adding SDS-PAGE loading buffer and heated at 100°C for 5 min. The reaction mixture was then subjected to an SDS-PAGE analysis.

### Pull-down assay

GST pulldown assays were performed^34^. Briefly, streptavidin, S, or glutathione agarose beads were incubated with cell lysates or purified proteins overnight. The beads were then washed with lysis buffer for five times.

### Immunoprecipitation and immunoblotting analysis

Proteins were extracted from cultured cells using a modified buffer, followed by immunoprecipitation and immunoblotting with the corresponding antibodies^35^. Each experiment was repeated at least three times. Full scans of immunoblotting are presented in Supplementary Fig. 8.

### Mass spectrometry analysis

An in vitro AKT1-phosphorylated purified PFKP was digested in-gel in 50 mM ammonium bicarbonate buffer containing Rapigest (Waters Corp., Milford, MA) overnight at 37°C with 200 ng of sequencing-grade modified trypsin (Promega, Madison, WI). The digest was analyzed by LC-MS/MS on an Obitrap-Elite mass spectrometer (Thermo Fisher Scientific, Waltham, MA). Proteins were identified by searching for the fragment spectra in the Swiss-Prot protein database (EBI) using the Mascot search engine (version 2.3; Matrix Science, London, UK) and SEQUEST v.1.27 (University of Washington, Seattle, WA) via the Proteome Discoverer software program (version 1.4; Thermo Fisher Scientific). Phosphopeptide matches were analyzed using the phosphoRS algorithm implemented in Proteome Discoverer and manually curated^36^.

### In vitro ubiquitylation assay

Purified WT HA-TRIM21 (2 μg) or HA-TRIM21 ΔRING mutant (2 μg) with purified His-PFKP were incubated with 50–500 nM E1, 0.5–5 μM His-E2 (Ubc4), 10 μM GST-Ub, and 2 mM ATP in a reaction buffer (50 mM Tris-HCl, pH 7.5, 2.5 mM MgCl_2_, and 0.5 mM DTT) for 90 min at room temperature.

### In vivo ubiquitylation assay

Cells were transfected with the indicated plasmids for 48 h and lysed using the denatured buffer (6 M guanidine-HCl [pH 8.0], 0.1 M Na_2_HPO_4_/NaH_2_PO_4_, and 10 mM imidazole) containing 5 mM N-ethylmaleimide to prevent deubiquitylation. The cell lysates were immunoprecipitated using the indicated antibodies, washed, and subjected to immunoblotting analysis.

### Metabolic assays

PFK and PK activity was determined using a PFK and PK activity colorimetric assay kit (BioVision, Milpitas, CA) following the standard protocols, respectively. The levels of glucose and lactate in cells were determined as described previously^20^. Glucose levels were determined using a glucose assay kit (Sigma). Glucose consumption was defined as the difference in glucose concentration compared with DMEM. Lactate levels were determined using a lactate assay kit (Eton Bioscience, San Diego, CA).

### Cell proliferation assay

A total of 2 × 10^4^ cells was plated and counted 5 days after being seeded in DMEM with 0.5% bovine calf serum. Data represent the means ± s.d. of three independent experiments.

### Intracranial implantation of GBM cells in mice

We injected 5 × 10^5^ U87/EGFRvIII GBM cells (in 5 μl of DMEM per mouse), with or without modulation of PFKP expression, intracranially into 4-week-old male athymic Balb/c nude mice (five mice/group). The injections were performed as described in a previous publication^20^. The mice were euthanized 2 weeks after the GBM cells had been injected. The brain of each mouse was harvested, fixed in 4% formaldehyde, and embedded in paraffin. Paraffin-embedded sections of mouse tumor tissues were prepared as described previously^20^. The sections were stained with hematoxylin-and-eosin (Biogenex Laboratories, San Ramon, CA) to determine tumor formation and phenotype. The slides were then mounted with Universal Mount (Research Genetics, Huntsville, AL).

All of the mice housed in the MD Anderson Cancer Center (Houston, Texas) animal facility, and all experiments were performed in accordance with relevant institutional and national guidelines and regulations approved by the Institutional Animal Care and Use Committee at MD Anderson Cancer Center.

### IHC analysis and scoring

An IHC analysis of Ki-67 was conducted using paraffin-embedded tissue sections. Ki-67 was detected with a VECTASTAIN Elite ABC kit (Vector Laboratories); tissue sections were then incubated with 3,3′-diaminobenzidine (Vector Laboratories), and the nuclei were stained with hematoxylin. Ki-67 staining was quantified by the percentage of positively stained nuclei per ×400 field. Six randomly chosen fields per slide were analyzed and averaged.

The human GBM samples and clinical information were from the Chinese Glioma Genome Atlas (CGGA, http://www.cgga.org.cn). This study was approved by the Ethics Committee of Capital Medical University (China), and written informed consents were obtained from all patients. The tissue sections from 65 paraffin-embedded human GBM specimens were stained with antibodies against phospho-AKT S473, phospho-PFKP S386, PFKP, or non-specific immunoglobulin as a negative control. We quantitatively scored the tissue sections according to the percentage of positive cells and staining intensity, as previously defined (Ji et al., 2009). We assigned the following proportion scores: 0 if 0% of the tumor cells showed positive staining, 0.1–1.0 if 0.1% to 1% of cells were stained, 1.1–2.0 if 1.1% to 10% stained, 2.1–3.0 if 11% to 30% stained, 3.1–4.0 if 31% to 70% stained, and 4.1–5.0 if 71% to 100% stained. We rated the intensity of staining on a scale of 0 to 3: 0, negative; 1, weak; 2, moderate; and 3, strong. We then combined the proportion and intensity scores to obtain a total score (range, 0–8), as described previously (Ji et al., 2009). Scores were compared with overall survival duration, defined as the time from the date of diagnosis to death or last known date of follow-up. The use of human glioblastoma samples and the clinical parameters was approved by the Institutional Review Board at Capital Medical University in Beijing, China.

### Tissue microarray analysis

A paraffin-embedded GBM tissue microarray was obtained from US Biomax (Rockville, MD), and all tissues are collected under the highest ethical standards with the donor being informed completely and with their consent. An IHC analysis of PFKP expression was performed according to the protocol described above.

### Statistical analysis

All quantitative data were presented as the mean ± s.d. of at least three independent experiments. A two-group comparison was conducted using a two-sided, two-sample Student’s *t*-test. A simultaneous comparison of more than 2 groups was conducted using one-way ANOVA (SPSS statistical package, version 12; SPSS Inc.). Values of *P* < 0.05 were considered statistically significant.

### Data availability

The clinical information related to the human GBM samples used in this study is available through the Chinese Glioma Genome Atlas (CGGA, http://www.cgga.org.cn). Data supporting the findings of this study are available within the article and from the authors upon reasonable request.

## Electronic supplementary material

Supplementary Information
