# Supplementary material for: Stabilization of phosphofructokinase 1 platelet isoform by AKT promotes tumorigenesis
Source: Nat Commun. 2017 Oct 16;8:949. doi: 10.1038/s41467-017-00906-9 (PMC5643558; doi:10.1038/s41467-017-00906-9)
Supplement: Supplementary file 1 — Supplementary Information [file 41467_2017_906_MOESM1_ESM.pdf]

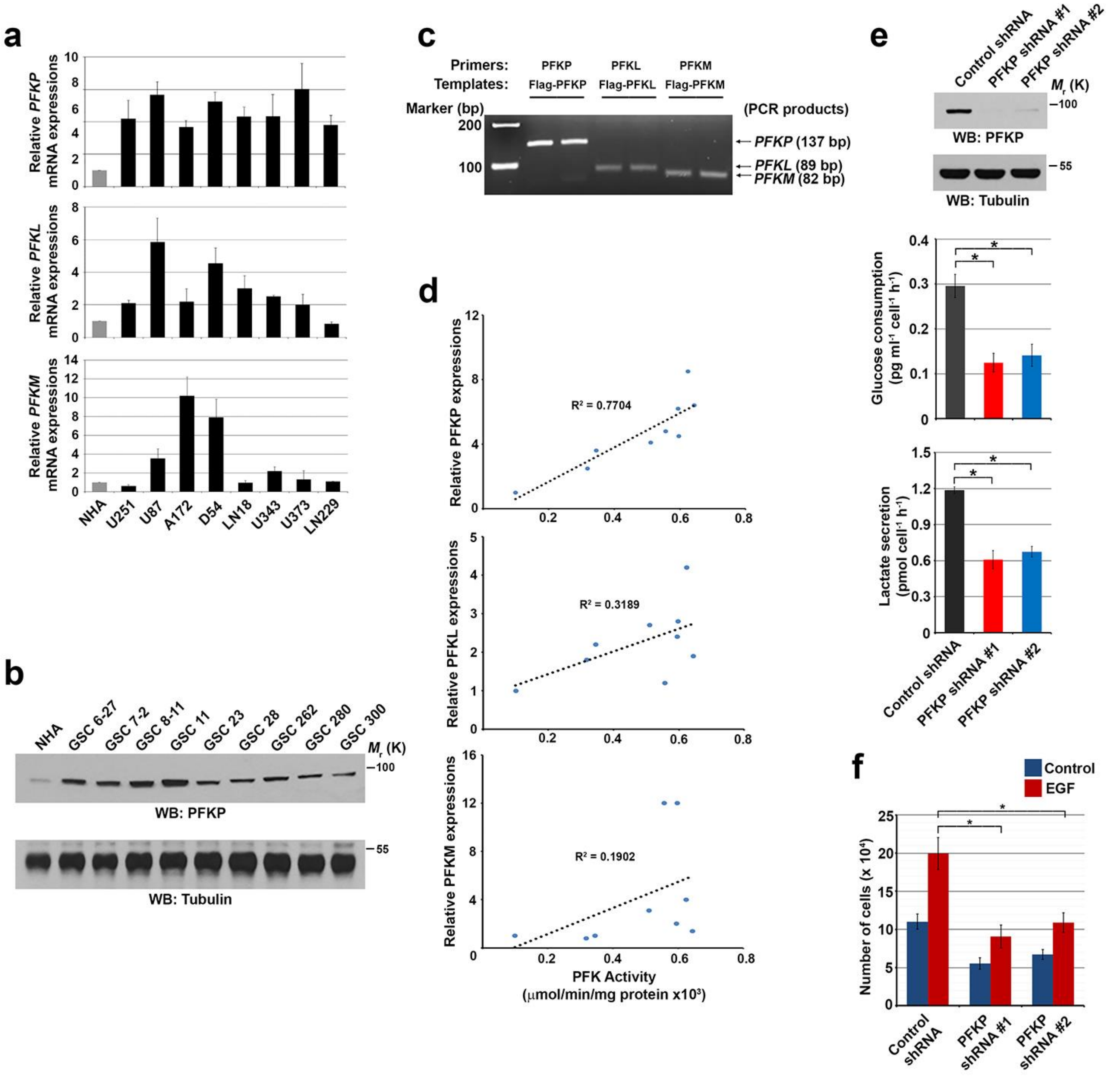

Supplementary Figure 1 PFKP expression is required for the Warburg effect and brain tumor growth.

(**a**) The mRNA expression levels of PFK1 isoforms in NHAs and the indicated GBM cells were determined by real-time PCR.

(**b**) PFKP expression was determined in NHAs and the indicated primary GBM cells. Immunoblotting analyses were performed with the indicated antibodies.

(**c**) RT-PCR products using primers against equal amounts of targeted plasmids (pcDNA3.1-Flag-PFKP, PFKL, or PFKM) were used to normalize the results of real-time PCR, as shown in Supplementary Fig. 1a. Each RT-PCR reaction was duplicated.

(**d**) The correlation between PFK activity (as shown in Fig. 1a) and protein levels (as shown in Fig. 1b) of the indicated PFK1 isoforms in NHAs and the indicated GBM cells was determined.

(**e**) U251 cells were transfected with different shRNAs against PFKP (top panel). The cells were cultured in no-serum DMEM for 24 h. The media were collected to analyze glucose consumption (middle panel) or lactate secretion (bottom panel). All results were normalized to the final cell number. Data represent the means ± SD of 3 independent experiments. $^*P < 0.001$, based on the Student's *t* test.

(**f**) U251 cells, with or without PFKP shRNA expression, were cultured in 1% serum medium, with or without EGF (100 ng/ml), for 4 days. The cells were collected and counted. Data represent the means ± SD of 3 independent experiments. $^*P < 0.001$, based on the Student's *t* test.

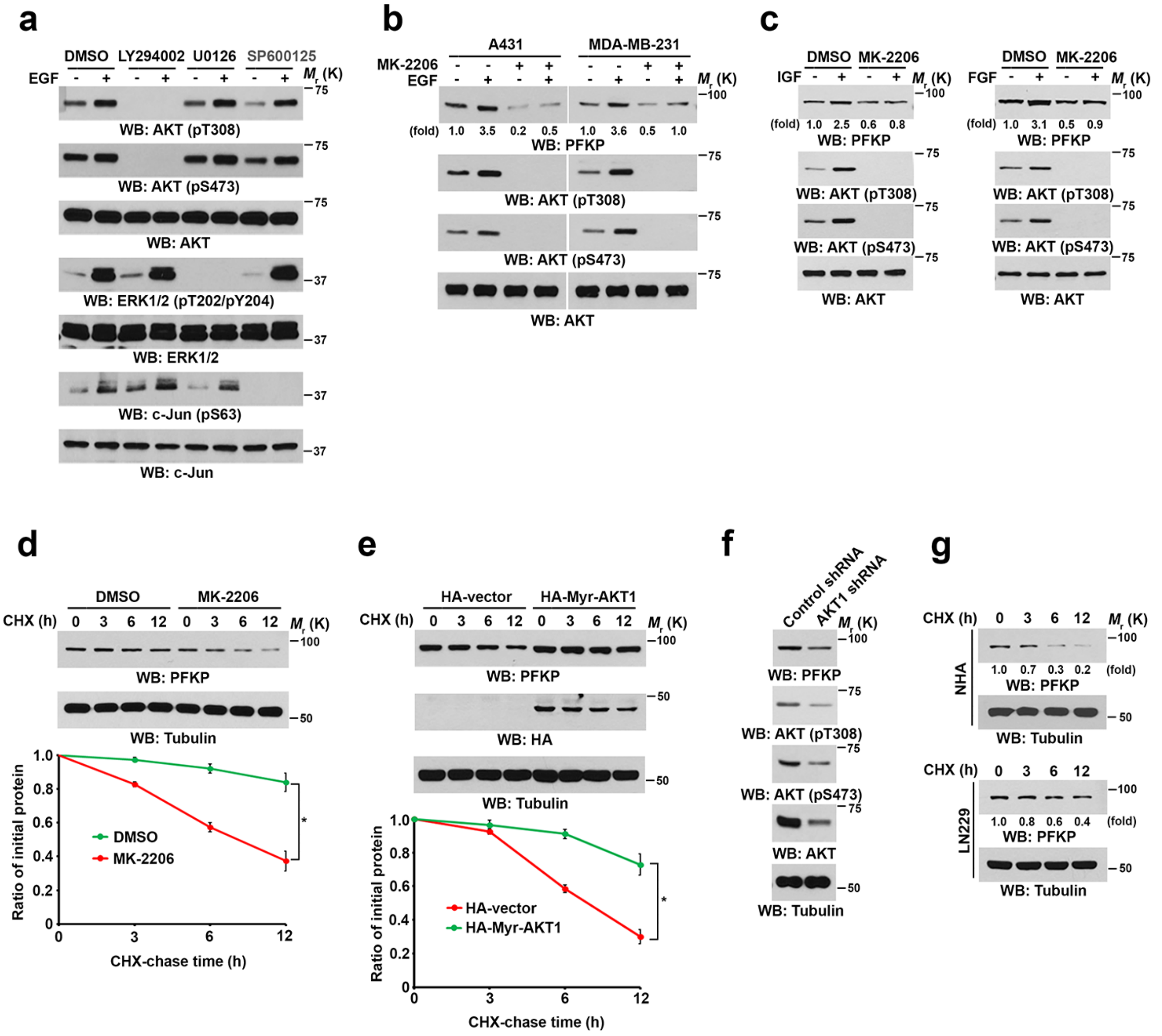

Supplementary Figure 2 AKT activation resulted from PTEN loss and EGFR-dependent PI3K activation induced PFKP upregulation.

Immunoblotting analyses were performed with the indicated antibodies.

(**a**) Serum-starved U251 cells were pretreated with the indicated inhibitors for 2 h and then stimulated with or without EGF (100 ng/ml) for 60 min.

(**b**) Serum-starved A431 and MDA-MB-231 cells were pretreated with DMSO or MK-2206 (5 μM) for 2 h and then stimulated with or without EGF (100 ng/ml) for 24 h.

(**c**) Serum-starved U251 cells were pretreated with DMSO or MK-2206 (5 μM) for 2 h and then stimulated with or without IGF (50 ng/ml) or FGF (50 ng/ml) for 24 h.

(**d**) U87/EGFRvIII cells were pretreated with DMSO or MK-2206 (5 μM) for 2 h and then treated with CHX (100 μg/ml) for the indicated periods of time. Quantification of PFKP levels relative to tubulin levels is shown. Data represent the means ± SD of 3 independent experiments. $*P < 0.01$, based on the Student's *t* test.

(**e**) 293T cells were transfected with HA-tagged control vector or HA-Myr-AKT1 and then treated with CHX (100 μg/ml) for the indicated periods of time. Quantification of PFKP levels relative to tubulin levels is shown. Data represent the means ± SD of 3 independent experiments. $*P < 0.01$, based on the Student's *t* test.

(**f**) U87/EGFRvIII cells with stable expression of AKT1 shRNA or a control shRNA were cultured in non-serum DMEM for 24 h.

(**g**) NHA and LN229 cells were treated with CHX (100 μg/ml) for the indicated periods of time.

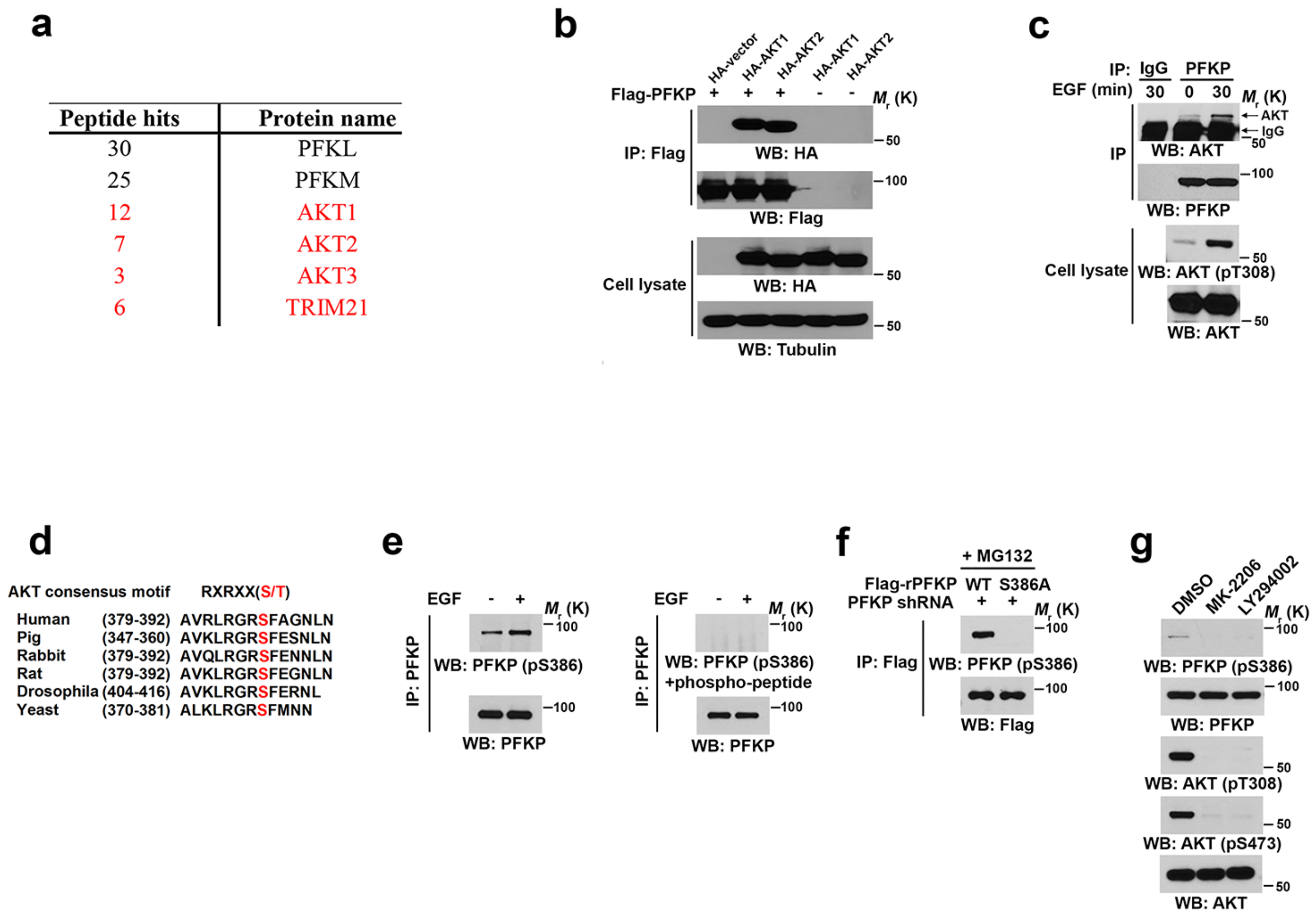

Supplementary Figure 3 AKT binds with and phosphorylates PFKP at Ser386.

Immunoblotting analyses were performed with the indicated antibodies (**b**, **c**, **e-g**).

(**a**) Selected peptide hits of PFKP-associated proteins in U251 cells, as identified by mass spectrometry, are presented. AKT1, AKT2, AKT3, and TRIM21 are highlighted.

(**b**) 293T cells were transfected with the indicated plasmids. Immunoprecipitation analyses were performed with an anti-Flag antibody.

(**c**) Serum-starved U87/EGFR cells were stimulated with or without EGF (100 ng/ml) for 30 min. Immunoprecipitation analyses were performed with an anti-PFKP antibody.

(**d**) S386 in human PFKP protein is conserved in other species. Serine residues are highlighted in red.

(**e**) Serum-starved U251 cells were stimulated with or without EGF (100 ng/ml) for 60 min. Immunoprecipitation with an anti-PFKP antibody was followed by immunoblotting analyses with an anti-phospho-PFKP S386 antibody in the presence or absence of specific blocking phosphopeptides.

(**f**) PFKP-depleted U87/EGFRvIII cells were reconstituted with WT Flag-rPFKP or Flag-rPFKP S386A mutant. MG132 (10 μM) was added to the cells 6 h before harvesting to eliminate the potential effect of proteasomal degradation on PFKP proteins. An immunoprecipitation analysis was performed.

(**g**) Serum-starved U87/EGFRvIII cells were treated with DMSO, MK-2206 (5 μM), or LY294002 (20 μM) for 2 h.

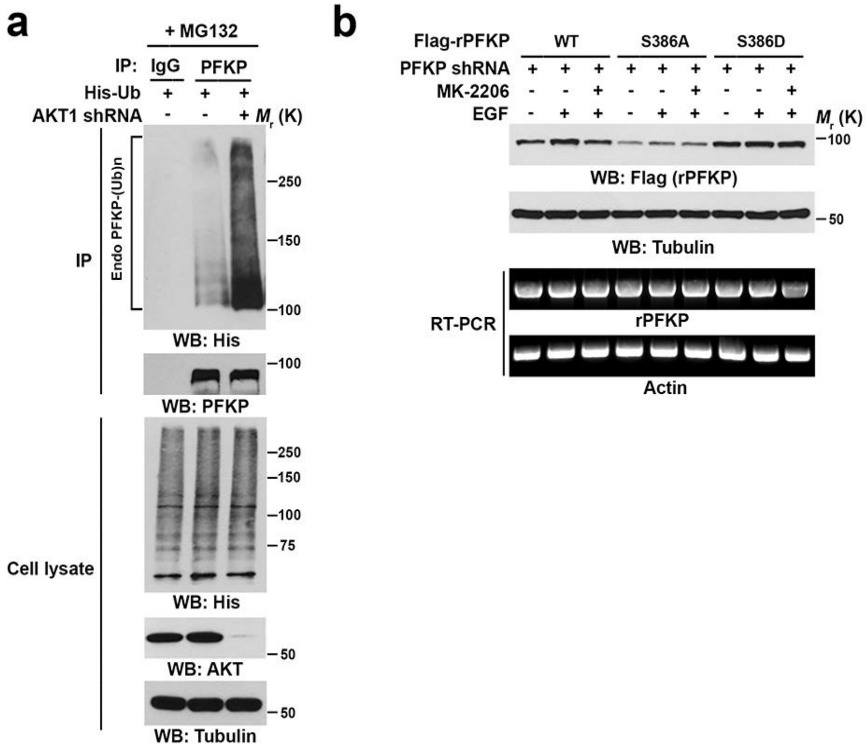

Supplementary Figure 4 AKT-dependent PFKP S386 phosphorylation blocks the ubiquitylation and degradation of PFKP.

Immunoblotting analyses were performed with the indicated antibodies

(**a**) U87/EGFRvIII cells with stable expression of AKT1 shRNA or a control shRNA were transfected with HA-Ub. MG132 (10 μM) was added to the cells 6 h before they were harvested with a guanidine-HCl-containing buffer. Immunoprecipitation with an anti-PFKP antibody was performed.

(**b**) Endogenous PFKP-depleted U251 cells with reconstituted expression of WT Flag-rPFKP, Flag-rPFKP S386A, or Flag-rPFKP S386D were pretreated with or without MK-2206 (5 μM) for 2 h and then stimulated with or without EGF (100 ng/ml) for 24 h. RT-PCR was performed with the indicated primers to show relatively equal expression of the indicated mRNAs.

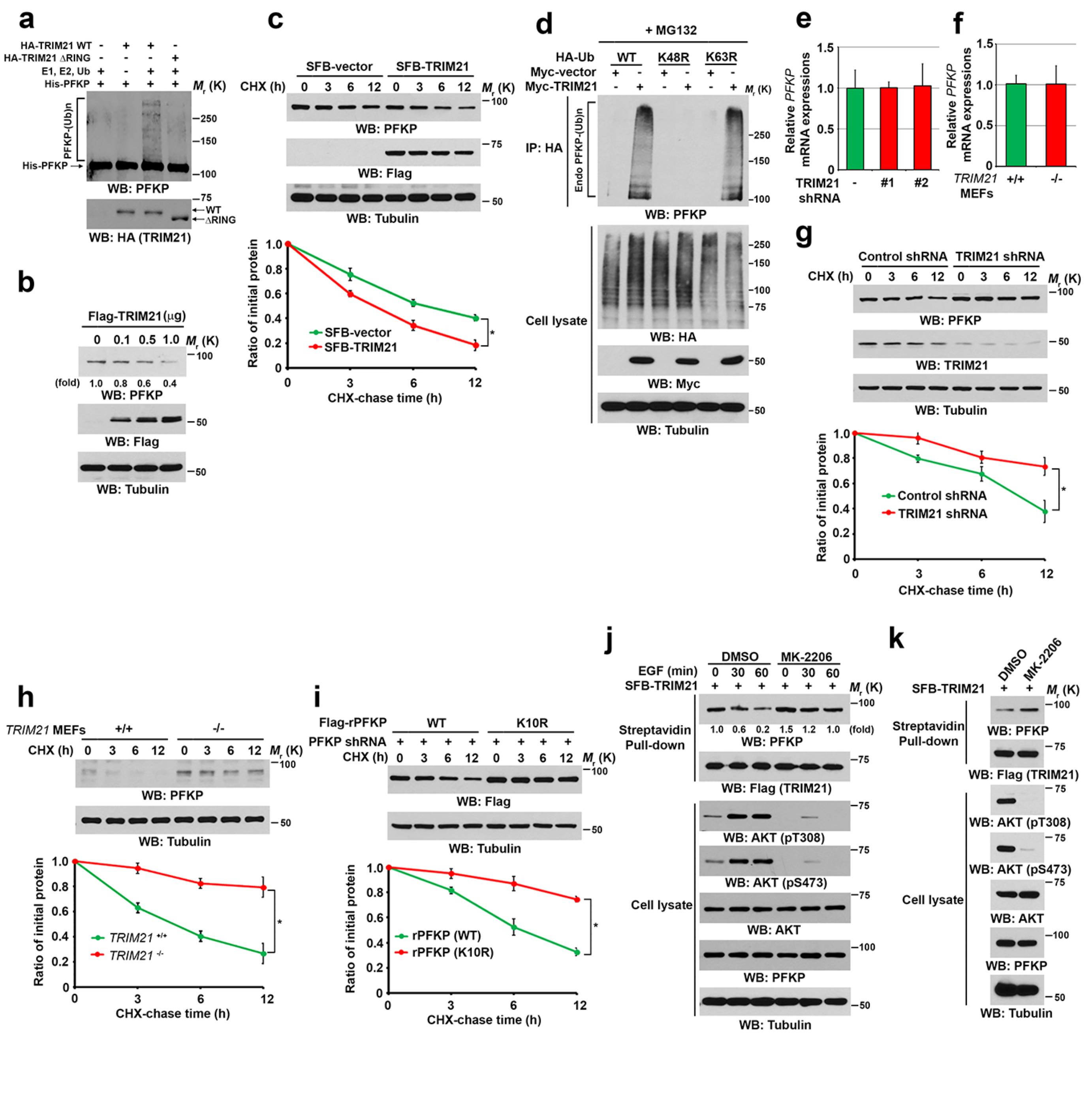

Supplementary Figure 5 TRIM21 mediates the polyubiquitylation and degradation of PFKP. Immunoblotting analyses were performed with the indicated antibodies (**a-d**, **g-k**).

(**a**) Purified His-PFKP was incubated with or without purified WT HA-TRIM21 or HA-TRIM21 ΔRING mutant in the presence or absence of purified E1, E2 (Ubc4), and ubiquitin.

(**b**) 293T cells were transfected with the indicated amounts of Flag-TRIM21.

(**c**) 293T cells were transfected with SFB-control vector or SFB-TRIM21 for 48 h and then treated with CHX (100 μg/ml) for the indicated periods of time. Quantification of PFKP levels relative to tubulin is shown. *$P < 0.05$, based on the Student's *t* test.

(**d**) 293T cells were co-transfected with HA-Ub WT or, K48R, K63R mutants and Myc-TRIM21. MG132 (10 μM) was added to the cells 6 h before they were harvested with a guanidine-HCl-containing buffer. Immunoprecipitation was performed with an anti-HA antibody.

(**e**) 293T cells were stably expressed with the TRIM21 shRNAs or a control shRNA. A real-time PCR analysis was performed.

(**f**) An analysis of PFKP mRNA levels in $TRIM21^{+/+}$ and $TRIM21^{-/-}$ MEF cells was performed.

(**g**) 293T cells with stable expression of TRIM21 shRNA or a control shRNA were treated with CHX (100 μg/ml) for the indicated periods of time. Quantification of PFKP levels relative to tubulin is shown. Data represent the means ± SD of 3 independent experiments. *$P < 0.01$, based on the Student's *t* test.

(**h**) $TRIM21^{+/+}$ and $TRIM21^{-/-}$ MEF cells were treated with CHX (100 μg/ml) for the indicated periods of time. Quantification of PFKP levels relative to tubulin is shown. Data represent the means ± SD of 3 independent experiments. *$P < 0.01$, based on the Student's *t* test.

(**i**) PFKP-depleted 293T cells were reconstituted with WT Flag-rPFKP or Flag-rPFKP K10R mutant and then treated with CHX (100 μg/ml) for the indicated periods of time. Quantification of Flag (rPFKP) levels relative to tubulin is shown. Data represent the means ± SD of 3 independent experiments. *$P < 0.01$, based on the Student's *t* test.

(**j**) SFB-TRIM21-expressing U251 cells were pretreated with DMSO or MK-2206 (5 μM) for 2 h and then stimulated with or without EGF (100 ng/ml) for the indicated periods of time. A pull-down assay was performed.

(**k**) U87/EGFRvIII cells were transfected with SFB-TRIM21 and then treated with DMSO or MK-2206 (5 μM) for 2 h. A pull-down assay was performed.

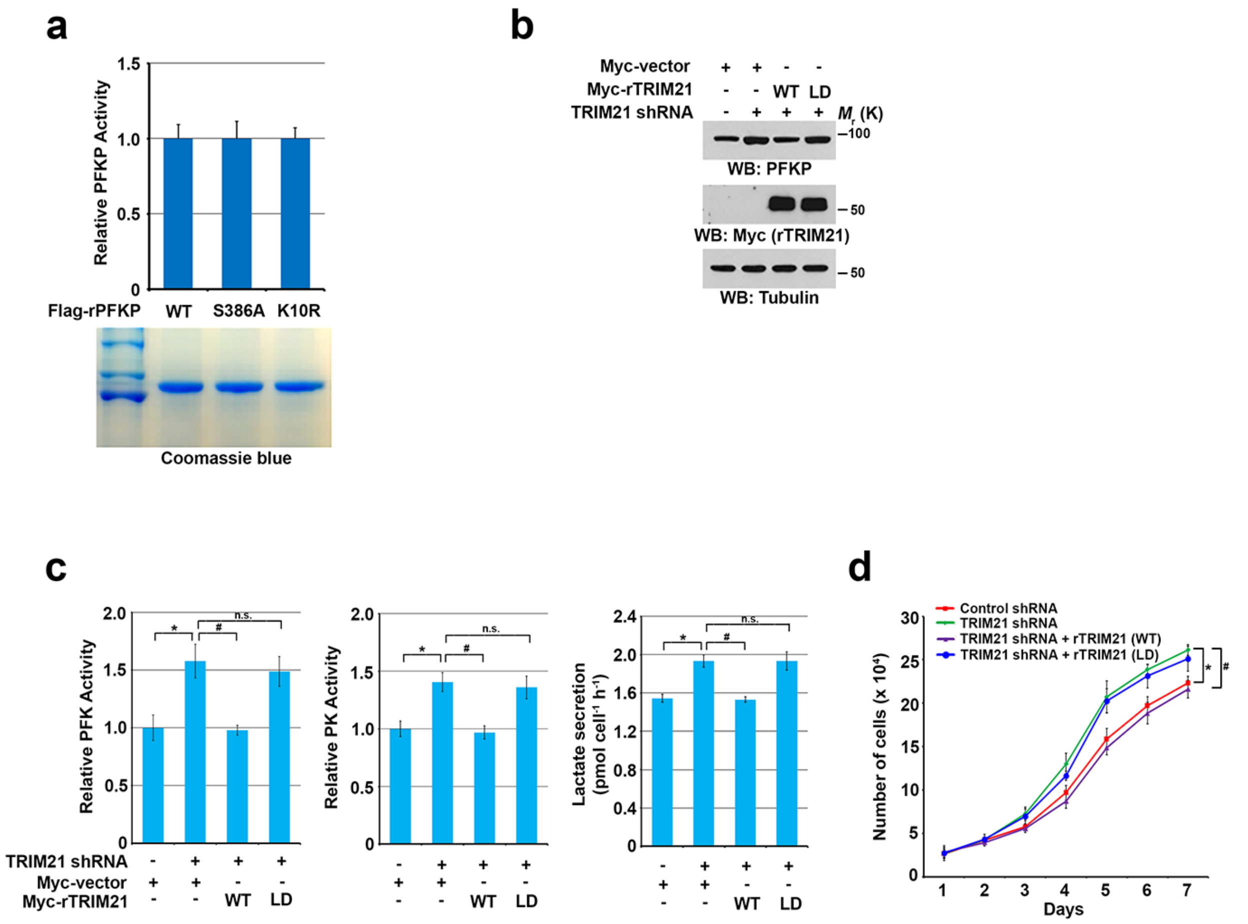

Supplementary Figure 6 PFKP S386 phosphorylation promotes glycolysis, cell proliferation, and tumor growth.

(**a**) PFKP-depleted 293T cells were transfected with the indicated plasmids for 48 h. The PFKP enzymatic activity of purified WT Flag-rPFKP, Flag-rPFKP S386A mutant, and Flag-rPFKP K10R mutant was measured. Data represent the means ± SD of 3 independent experiments.

(**b**) U87/EGFRvIII cells with or without TRIM21 depletion were reconstituted with WT Myc-rTRIM21 or Myc-rTRIM21 LD mutant. Immunoblotting analyses were performed with the indicated antibodies.

(**c**) U87/EGFRvIII cells that stably expressed or did not express TRIM21 shRNA, with reconstituted expression of WT Myc-rTRIM21 or Myc-rTRIM21 LD mutant, were cultured in non-serum DMEM for 24 h. The cells and the media were collected to analyze glucose consumption, PFK activity, PK activity, or lactate secretion. All results were normalized to the final cell number. Data represent the means ± SD of 3 independent experiments. $^{*}P < 0.01$, $^{\#}P < 0.01$, based on the one-way ANOVA; n.s., not significant.

(**d**) U87/EGFRvIII cells with or without TRIM21 depletion and reconstituted expression of WT Myc-rTRIM21 or Myc-rTRIM21 LD mutant were cultured in 1% serum medium for the indicated periods of time and harvested for cell counting. Data represent the mean ± SD of 3 independent experiments. $^{*}P < 0.01$, $^{\#}P < 0.01$, based on the Student's *t* test.

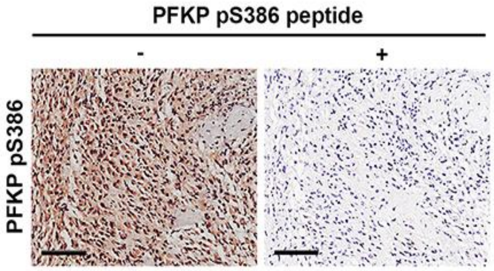

Supplementary Figure 7 AKT pS473 expression is positively correlated with PFKP pS386 or PFKP expression in human GBM. Antibody specificity was validated in human GBM specimens, in the presence or absence of a blocking peptide that was specific for phosphorylated PFKP S386. Scale bar, 100 μm.

Figure 1b

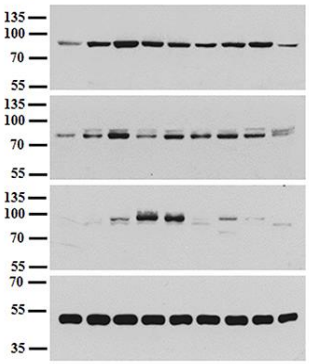

Figure 1e

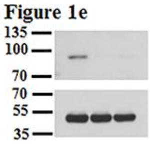

Figure 2a

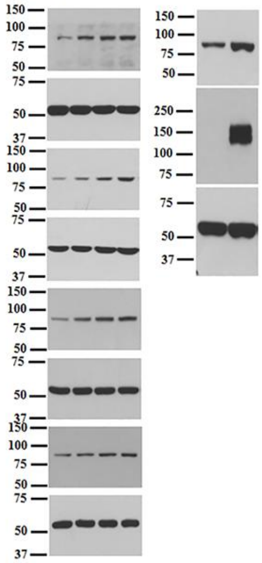

Figure 2b

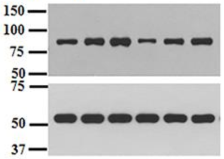

Figure 2c

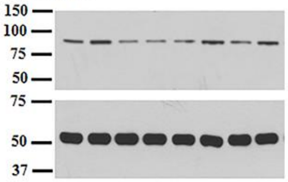

Figure 2d

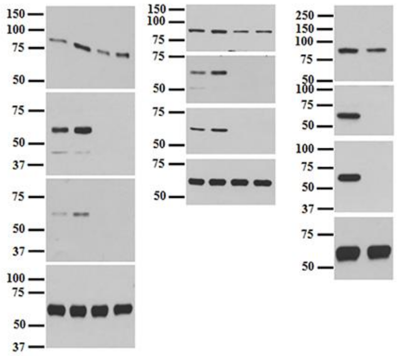

Figure 2e

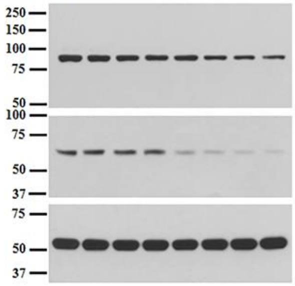

Figure 2f

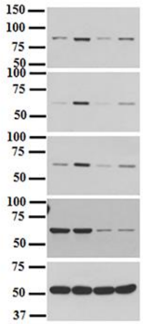

Figure 2g

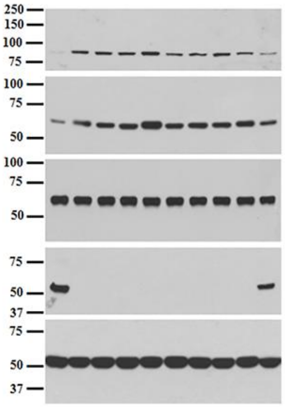

Figure 2h

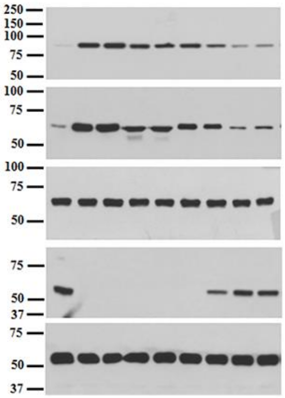

Figure 2i

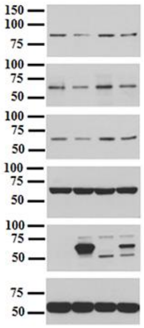

Figure 3a

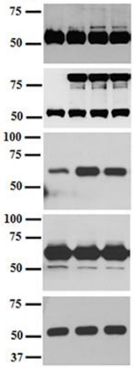

Figure 3b

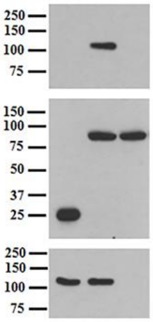

Figure 3c

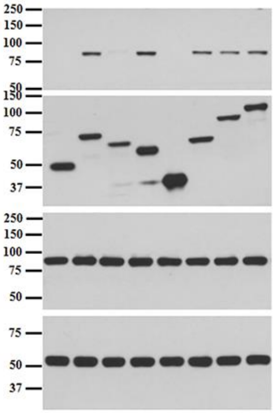

Figure 3f

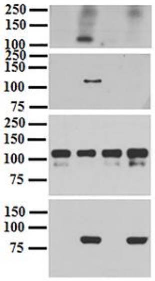

Figure 3g

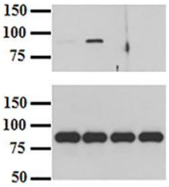

Figure 3h

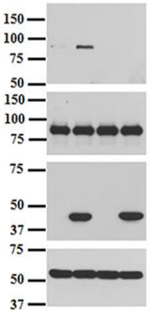

Figure 3i

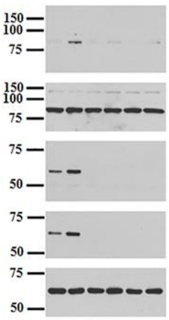

Figure 4a

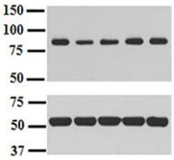

Figure 4b

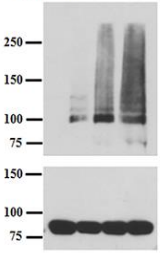

Figure 4c

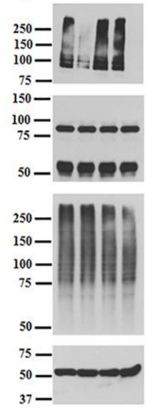

Figure 4d

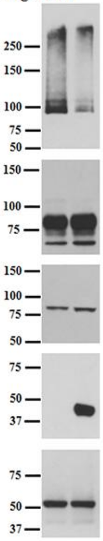

Figure 4e

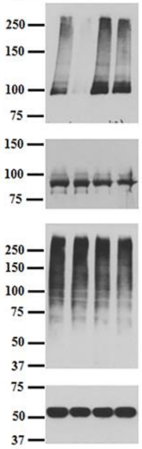

Figure 4f

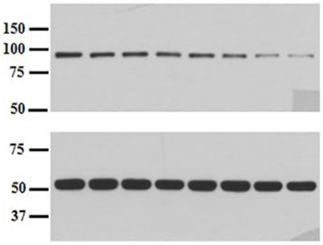

Figure 4g

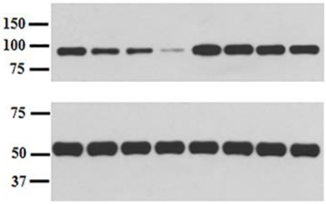

Figure 5a

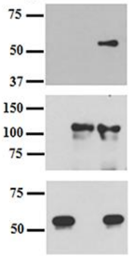

Figure 5b

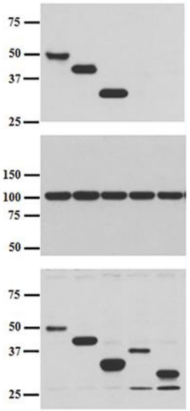

Figure 5c

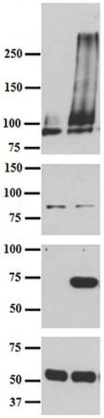

Figure 5d

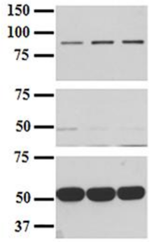

Figure 5e

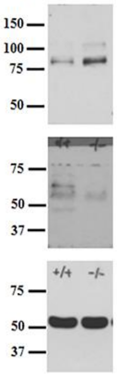

Figure 5f

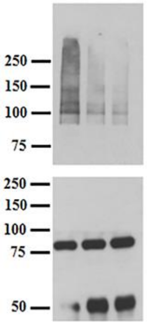

Figure 5g

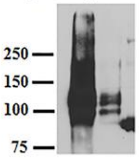

Figure 5h

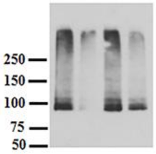

Figure 5i

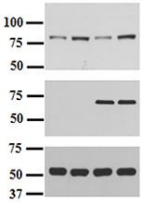

Figure 5j

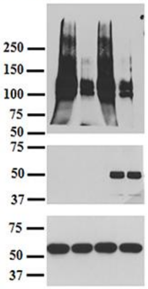

Figure 5k

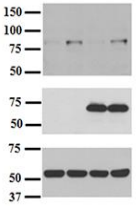

Figure 5l

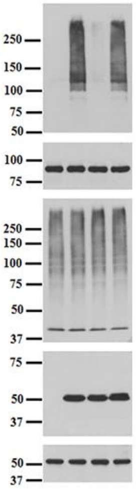

Figure 5m

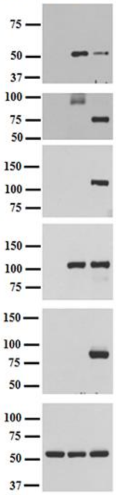

Figure 5n

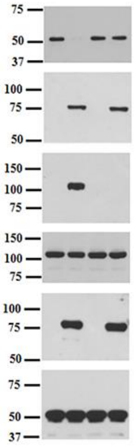

Figure 5o

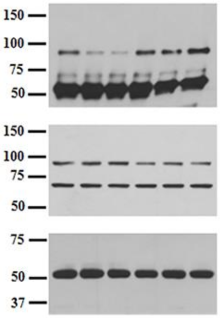

Figure 5p

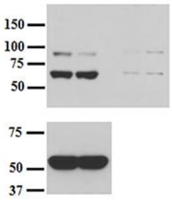

Figure 6a

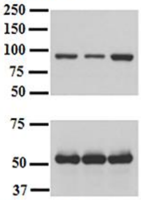

Figure S1b

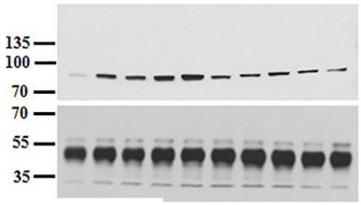

Figure S1e

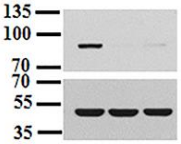

Figure S2a

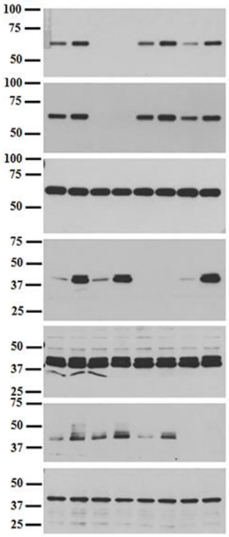

Figure S2b

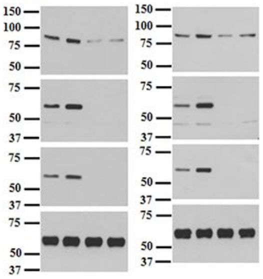

Figure S2c

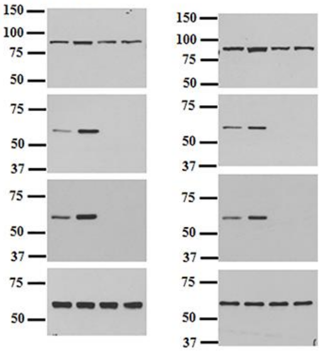

Figure S2d

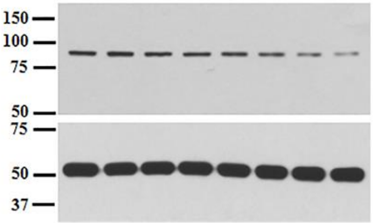

Figure S2e

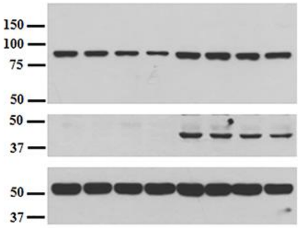

Figure S2f

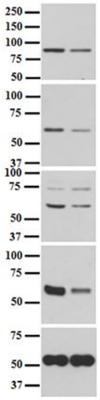

Figure S2g

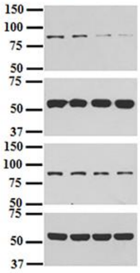

Figure S3b

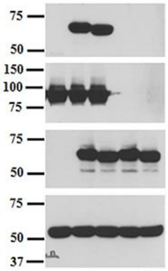

Figure S3c

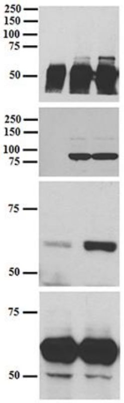

Figure S3e

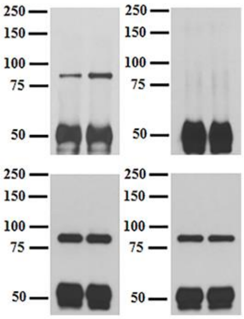

Figure S3f

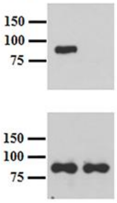

Figure S3g

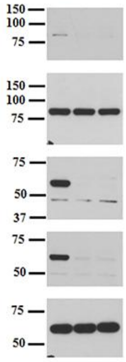

Figure S4a

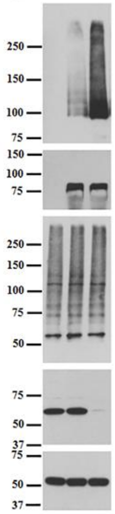

Figure S4b

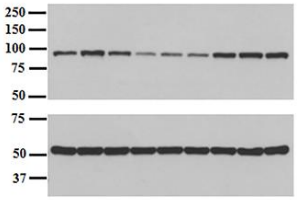

Figure S5a

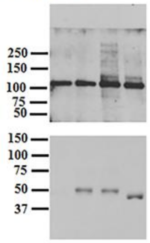

Figure S5b

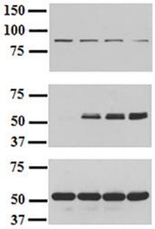

Figure S5c

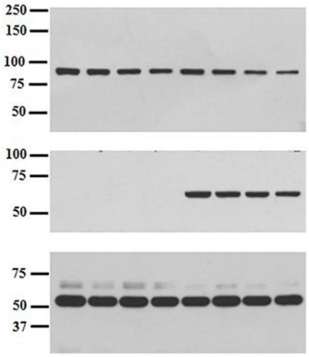

Figure S5d

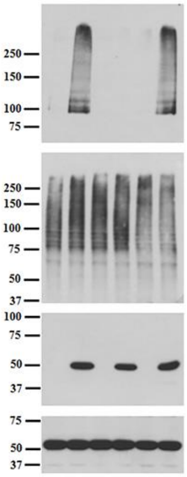

Figure S5g

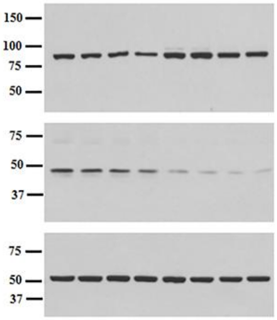

Figure S5h

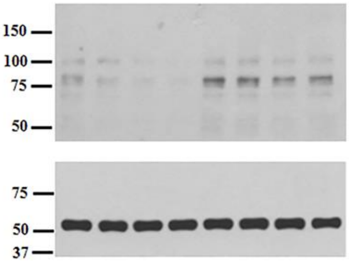

Figure S5i

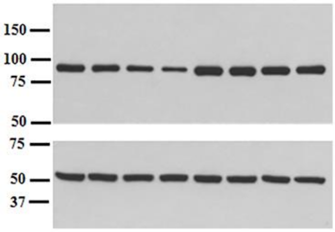

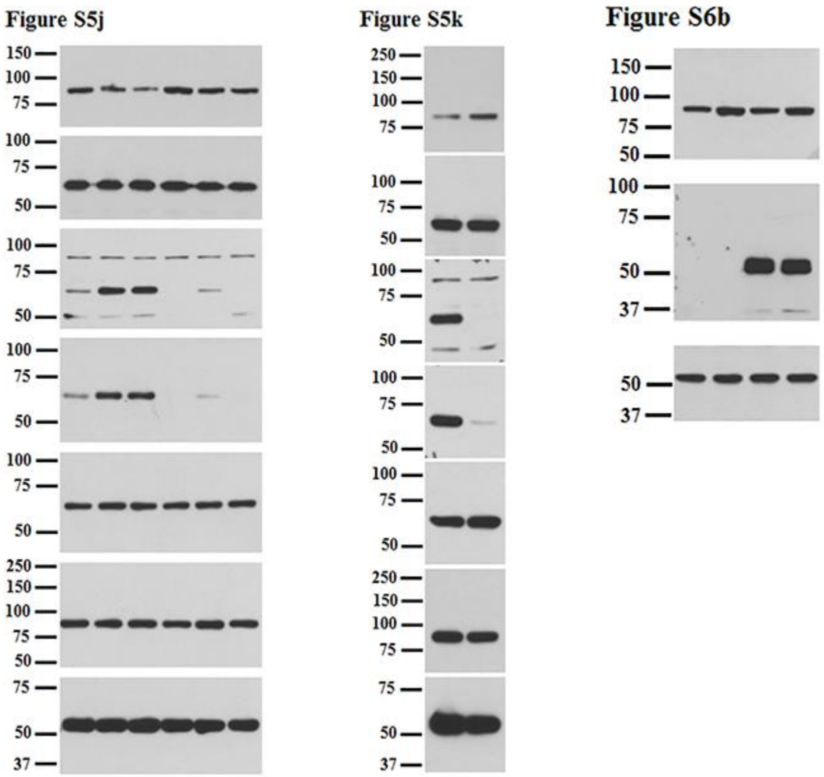

Supplementary Figure 8 The unprocessed blots.

PFKP Ubiquitination Site

MDADDSRAPKGSLRKFLEHLSGAGKAIGVLTSGGDAQGMNAAVRAVVRMGIYVGAKVYFIYEGYQGMVDG
GSNIAEADWESVSSILQVGGTIIGSARCQAFRTREGRLKAACNLLQRGITNLCVIGGDGSLTGANLFRKE
WSGLLEELARNGQIDKEAVQKYAYLNVVGMVGSIDNDFCGTDMTIGTDSALHRIIEVVDAIMTTAQSHQR
TFVLEVMGRHCGYLALVSALACGADWVFLPESPPEEGWEEQMCVKLSENRARKKRLNIIIVAEGAIDTQN
KPITSEKIKELVVTQLGYDTRVTILGHVQRGGTPSAFDRILASRMGVEAVIALLEATPDTPACVVSLNGN
HAVRLPLMECVQMTQDVQKAMDERRFQDAVRLRGRSFAGNLNTYKRLAIKLPDDQIPKTNCNVAVINVGA
PAAGMNAAVRSAVRVGIADGHRMLAIYDGFDGFAKGQIKEIGWTDVGGWTGQGGSILGTKRVLPGKYLEE
IATQMRTHSINALLIIGGFEAYLGLLELSAAREKHEEFCVPMVMVPATVSNNVPGSDFSIGADTALNTIT
DTCDRIKQSASGTKRRVFIIETMGGYCGYLANMGGLAAGADAAYIFEEPFDIRDLQSNVEHLTEKMKTTI
QRGLVLRNESCSENYTTDFIYQLYSEEGKGVFDCRKNVLGHMQQGGAPSPFDRNFGTKISARAMEWITAK
LKEARGRGKKFTTDDSICVLGISKRNVIFQPVAELKKQTDFEHRIPKEQWWLKLRPLMKILAKYKASYDV
SDSGQLEHVQPWSV

Output:

Residue	Score	Ubiquitinated		Residue	Score	Ubiquitinated
10	0.74	Yes Medium confidence		524	0.42	No
15	0.33	No		567	0.20	No
25	0.58	No		574	0.08	No
56	0.43	No		625	0.43	No
109	0.18	No		627	0.21	No
139	0.42	No		659	0.59	No
156	0.53	No		666	0.49	No
161	0.45	No		688	0.30	No
255	0.48	No		700	0.31	No
263	0.19	No		702	0.25	No
264	0.14	No		709	0.19	No
281	0.59	No		710	0.18	No
287	0.39	No		724	0.42	No
289	0.44	No		736	0.35	No
395	0.39	No		737	0.48	No
400	0.48	No		747	0.30	No
408	0.56	No		753	0.15	No
455	0.32	No		759	0.13	No
459	0.38	No		763	0.15	No
480	0.40	No		765	0.45	No
486	0.38	No				

Legend:

Label	Score range	Sensitivity	Specificity
Low confidence	0.62 ≤ s ≤ 0.69	0.464	0.903
Medium confidence	0.69 ≤ s ≤ 0.84	0.346	0.950
High confidence	0.84 ≤ s ≤ 1.00	0.197	0.989

Supplementary Table 1 The potential ubiquitylation sites of PFKP are shown. The possibility of ubiquitylation of lysine residues in PFKP (middle panel) was predicted by analyses of the amino acid sequence of PFKP (top panel) by “UbPred: predictor of protein ubiquitination sites”.
